# A ‘choice’, an ‘addiction’, a way ‘out of the lost’: exploring self-injury in autistic people without intellectual disability

**DOI:** 10.1186/s13229-019-0267-3

**Published:** 2019-04-11

**Authors:** R. L. Moseley, N. J. Gregory, P. Smith, C. Allison, S. Baron-Cohen

**Affiliations:** 1 Social, Cognitive, Clinical and Affective Neuroscience group, Department of Psychology, Bournemouth University, Talbot Campus, Fern Barrow, Poole, Dorset UK; 2 Autism Research Centre, Department of Psychiatry, University of Cambridge, Cambridge, UK

**Keywords:** Self-injury, Self-harm, Autism, Alexithymia, Sensory differences, Suicidality, Qualitative

## Abstract

**Background:**

Non-suicidal self-injury (NSSI) describes a phenomenon where individuals inflict deliberate pain and tissue damage to their bodies. Self-injurious behaviour is especially prevalent across the autism spectrum, but little is understood about the features and functions of self-injury for autistic individuals without intellectual disability, or about the risk factors that might be valuable for clinical usage in this group.

**Methods:**

One hundred and three autistic adults who responded to an online advertisement were classified as current, historic or non-self-harmers in accordance with responses to the Non-Suicidal Self-Injury Assessment Tool (NSSI-AT). Multinomial regression aimed to predict categorisation of participants in accordance with scores on tests of autistic traits, alexithymia, depression, anxiety, mentalising and sensory sensitivity. Linear regression examined relationships between these predictors and the range, frequency, lifetime occurrence and functional purposes of NSSI. Qualitative analysis explored the therapeutic interventions that participants had found helpful, and what they wished people understood about self-injury.

**Results:**

Current, historic and non-self-harming participants did not differ in age, age at diagnosis, male-to-female ratio, level of employment or education (the majority qualified to at least degree level). The most common function of NSSI was the regulation of low-energy affective states (depression, dissociation), followed by the regulation of high-energy states such as anger and anxiety. Alexithymia significantly predicted the categorisation of participants as current, historic or non-self-harmers, and predicted use of NSSI for regulating high-energy states and communicating distress to others. Depression, anxiety and sensory-sensitivity also differentiated participant groups, and sensory differences also predicted the range of bodily areas targeted, lifetime incidence and frequency of NSSI. Sensory differences, difficulty expressing and identifying emotions also emerged as problematic in the qualitative analysis, where participants expressed the need for compassion, patience, non-judgement and the need to recognise diversity between self-harmers, with some participants perceiving NSSI as a practical, non-problematic coping strategy.

**Conclusions:**

Alexithymia, depression, anxiety and sensory differences may place some autistic individuals at especial risk of self-injury. Investigating the involvement of these variables and their utility for identification and treatment is of high importance, and the voices of participants offer guidance to practitioners confronted with NSSI in their autistic clients.

**Electronic supplementary material:**

The online version of this article (10.1186/s13229-019-0267-3) contains supplementary material, which is available to authorized users.

Non-suicidal self-injury (NSSI), also known as self-mutilation or self-harm, describes acts of purposeful, physical, sometimes painful damage to the body *without* suicidal intent [1]. These must be differentiated from what might be described as self-damaging or risky behaviours which afford some kind of pleasurable or anxiolytic effects despite known health risk (e.g. drinking, smoking, unsafe sex); cutting or burning of the skin are examples of self-injurious behaviour which reflect ‘attempts to modify one’s affective/cognitive or social experience’ [1] (p. 15.5). NSSI tends to begin in adolescence and is a common feature of a number of mental health conditions. It may serve a number of functional roles including emotion regulation (breaking through states of numbness, depression or dissociation or discharging ‘hot’ feelings of anger, frustration, agitation), self-punishment, sensory stimulation, as a means of communicating to or influencing others, or even in avoidance of more severe actions [2]. As NSSI is often associated with later suicide attempts [3–9], clinical and research attention to these behaviours is imperative.

One group at substantially higher risk of suicidality and mental illness are autistic people [10, 11]. Despite this, only recently have attempts been made to understand the occurrence and nature of non-suicidal self-injury in this group.^1^ NSSI in autism spectrum conditions (ASC) is challenging to define due to the presence of high-frequency self-injurious behaviours, such as head-banging and biting, which are commonly classed as ‘stereotyped’ [12], as elements of repetitive and restricted behaviours and interests (RRBI). These self-injurious behaviours, which have been the focus of autism research in children and adults [12–14], appear to differ in nature from NSSI in the typically developing populations: they occur in front of others without attempts to disguise them, being most commonly associated with intellectual disability and severe receptive and expressive language deficits. Maddox et al. [15] were recently the first to delineate a different type of NSSI, one resembling that seen in neurotypical populations, in autistic people without intellectual disability (all of whom had completed high school, the majority of whom had college qualifications). They found more similarities than differences between their small groups of autistic and non-autistic (typically developing) self-harmers; both began self-injuring in early adolescence and did not differ in the specific NSSI they engaged in. No significant differences were seen in their perceived reasons for NSSI: both groups were equally likely to engage in NSSI in attempts to modulate ‘low pressure’ emotions such as depression or dissociation, to release ‘high pressure’ emotions like agitation and anger, as a form of communication or social influence, to punish themselves or avoid more serious actions and consequences, and to seek simulation. The only suggested differences were that autistic participants were more likely than non-autistic self-harmers to engage in NSSI for the purpose of shocking or hurting others, in imitation of peers, or for the purpose of avoiding a suicide attempt. These authors also compared autistic self-harmers to autistic people who did not engage in NSSI: they found no significant differences in age, depression or emotion dysregulation, but a higher proportion of autistic women than autistic men engaged in NSSI.

This important paper was the first to explore autistic NSSI *not* within the RRBI domain, and *not* in individuals with intellectual impairment, but in individuals with IQ within the normal range and with consideration of the roles or functions of NSSI as reported within the typical population and other clinical groups. Crucially, it highlighted the increased prevalence of NSSI in autistic as compared with typically developing populations, with 50% of the autistic sample (*n* = 42) having engaged in at least one act of NSSI—an inflated prevalence that motivates further study of NSSI in a larger sample of these individuals within the autism spectrum.

In their analysis, Maddox et al. considered the association of depression and emotion dysregulation with NSSI by comparing these factors between autistic self-harmers and autistic non-self-harmers. They found no significant differences between groups and so suggest that these factors are not associated with increased risk of self-injury. We note, however, that the small sample did not allow the authors to differentiate between *current* and *historic* self-harmers within their NSSI group, instead categorising participants dichotomously based on lifetime incidence of NSSI. This dichotomous categorisation may have hidden group differences if, for example, current self-harmers suffer from greater depression and emotional dysregulation than those whose NSSI is in the past and who judge themselves unlikely to engage in these behaviours in future, and thus leaves open the question as to whether depression and emotional dysregulation are indeed risk factors for current engagement in NSSI.

In our consideration of potential factors of clinical relevance, the functions that NSSI serves in autistic (and typically developing) people afford vital clues for a theory-led analysis. The use of NSSI for emotion regulation, for example, does implicate current depression as a risk factor in participants who engage in NSSI to manage depressive or dissociative states [2]. NSSI is also employed for the management of high energy states such as anger, anxiety, frustration and agitation, where it seems to act as a kind of pressure valve. One common associate of NSSI that may be of relevance here is alexithymia, a difficulty understanding and identifying one’s own emotions and those of other people. Alexithymia is common in people who self-injure [16]: these individuals not only have difficulty expressing their emotions verbally [17], a key aspect of alexithymia, but tend to be less aware of their emotional states [18, 19]. Borrill et al. [20] identified alexithymia as a strong predictor of self-injury in a student sample, and noted that it was especially associated with repetitive NSSI. Others have identified alexithymia as an important mediator which increases the risk of NSSI in people who have experienced different types of life adversity, such as childhood abuse and bullying [16, 21–23]. In clinical groups, better ability to label and differentiate between negative emotions is associated with decreased likelihood of NSSI [24].

Another variable of potential relevance may relate to the function of NSSI as a means of communicating distress or anger to others [2]. Whilst the inability to verbalise one’s emotions is again of high relevance here, the use of self-injury as a means of communication when verbal means fail may reflect the interpersonal difficulties at the core of ASC. These communication difficulties have been linked to difficulties with ‘theory of mind’, also known as mentalising [25, 26], which is very important for effective communication. Whilst mentalising impairments or differences have not been previously linked to self-injury as such, they are a feature of borderline personality disorder [27–30], which is itself strongly linked with self-injury [31–33]. In this vein, some authors have hypothesised that mentalising deficits might lead to more intense negative feelings and social isolation, thus leading to manipulation and self-harm to create connection with others [34, 35]. Accordingly, interventions aiming to strengthen mentalising ability have been seen to decrease self-injury alongside borderline symptomatology [36, 37], with further trials ongoing [38]. This motivates investigation of mentalising impairments as predictive of self-injury in autism. The presence of mentalising impairments in autistic individuals, and the relationship between mentalising abilities and autistic traits [39–41], suggests that autistic symptom severity (in so far as it predicts mentalising impairment) could also be a predictor of NSSI. Indeed, the fact that autistic symptom severity predicts the severity and frequency of the ‘stereotyped’ forms of NSSI seen in adults with learning disabilities [42] motivates investigation of autistic symptom severity as a predictor of the form of NSSI described by Maddox et al.

In contrast to its role in communication, another potential variable of interest is highlighted by use of NSSI to generate sensory stimulation [2], with non-autistic self-harmers reporting a ‘rush’ or a ‘high’, a feeling of excitement, after engaging in NSSI [43, 44]. Therapeutic interventions may reroute this drive by providing alternative strategies for stimulation [45]. Sensory stimulation as a function of NSSI is highly relevant for autistic people, as different sensory-perceptual experience of the world is very common in autism (see [46, 47]). Some individuals show a pattern of low registration or under-responsivity (a weak response to stimulation due to a high neurological threshold [47]); some seek sensation for stimulation; others show sensory sensitivity or over-responsivity, a low neurological threshold leading to exaggerated and uncomfortable sensory experiences [47]; heterogeneous sensory symptoms are modulated by age, IQ and severity of autism, and individuals may show more than one pattern in different sensory modalities [48]. Importantly, sensory differences in autism are associated with ritualistic and repetitive behaviours including self-injury [49], and are even the strongest predictors of self-injury [13]. However, these studies have focused on self-injury of the type which Maddox et al. note is more characteristic of individuals with intellectual disability and language problems; consequently, investigation of whether sensory differences are also important for self-injury in individuals without intellectual disability is timely.

The aims of the present report are threefold: whilst aiming to validate Maddox et al.’s descriptive analysis of NSSI within a larger autistic population without intellectual disability, we further aimed to qualitatively analyse participants’ experiences of NSSI, and to explore predictive factors for NSSI that might thus be of clinical relevance. In a mixed methods approach, a descriptive report of the characteristics of autistic self-harmers and their self-injurious behaviour was bolstered by statistical regression in order to examine alexithymia, mentalising impairments, autistic traits and sensory differences as variables that might predict (a) the presence of self-injury; (b) the severity, range and frequency of these behaviours; and (c) the use of self-injury to meet certain functional purposes. To explore participants’ individual experience of NSSI, we employed the qualitative method of thematic analysis to examine participants’ responses to two open questions regarding their experience of therapeutic help and what they would like others to know about self-injury.

## Methods

### Participants

Autistic participants were recruited from support groups local to the primary researcher (Dorset) and via the Cambridge Autism Research Database (CARD) at the Autism Research Centre, Cambridge, UK. Details of the study were sent to 2264 national and international volunteers with a formal diagnosis of autism,^2^ and 103 participants took part in the study. The group (*n* = 70 females and *n* = 33 males) had an average age of 43 years old (SD = 13.6) and was diagnosed, on average, at 34.2 years old (SD = 16.2). The majority of them (66%) were British, but other nationalities included American, Australian, Hungarian, Finnish, Dutch, German, Swedish, Irish, Scottish, Italian, Canadian, New Zealandic, Czech and Venezualan. Although IQ was not measured, all participants had attended school to GCSE level (or equivalent) and the majority (64%) had degrees, such that it was possible to infer that participants did not have an intellectual impairment (IQ < 70). Just over half of the participants (52%) were employed. Forty-nine percent were taking some kind of psychotropic medication at the time of the study; 75% had been diagnosed with at least one comorbid psychiatric condition, the most common being depression and/or anxiety. Twenty-two participants had been diagnosed with a specific learning difficulty such as dyslexia or dyspraxia; 12 had been diagnosed with ADHD.

Participants were classified as current self-harmers (*n* = 49), historic self-harmers (*n* = 27) and non-self-harmers (*n* = 27) (see ‘Methods’ section): the demographic details of each group are given in the ‘Results’ section. Individuals (4) who responded to the advertisement but whose NSSI occurred in the context of suicide attempts were not included in this analysis.

## Materials

Variables of interest in relation to NSSI included alexithymia, autistic traits, sensory processing differences, mentalising abilities, depression and anxiety.

Alexithymia was measured with the Toronto Alexithymia Scale (TAS-20 [50]), a self-report measure which asks participants to rate their agreement with 20 items reflecting their recognition and understanding of their own emotional states, their ability to verbalise them to others and their tendency for externally orientated thinking. The authors report good internal consistency and test-retest reliability (alexithymia is understood as a stable construct), and the instrument has been translated and used across a substantial number of countries and cultures [51]. Whilst these authors found the three distinct factors named above to be largely reliable across cultures, other findings contradict this, especially in patient groups ([52, 53], though Loas et al. showed that this may depend on the patient group [54]). We used a single overall score from the TAS-20 to reflect degree of alexithymia, which encompasses all three of the above in its clinical presentation.

The Autism-Spectrum Quotient (AQ) [55] is a quantitative measure of autistic traits that can be used in the general population as well as in clinical groups. A recent systematic review revealed the extent of its usage and confirmed normative mean scores of 16.9 for typically developing and 35 for autistic people [56]. The test has good internal consistency and test-retest reliability and has been translated into many languages [57, 58]. Whilst it consists of five subscales (social skills, attention switching, attention to detail, communication and imagination), we used a single score to reflect autistic traits in each participant.

The Adolescent-Adult Sensory Profile [59] is based on Dunn’s [47] model of sensory processing. It measures scores in four domains: low registration (weak response to stimulation due to high neurological threshold), sensation seeking (a similar weak response to stimulation coupled with a drive to counter this), sensory sensitivity (a high response to sensation due to a low neurological threshold, manifest in distractibility and discomfort), and sensory avoidant (similar low threshold coupled with behaviours limiting exposure to stimuli). The test has good construct validity in terms of skin conductivity and good internal consistency [59], and is used clinically.

The ‘Reading the Mind in the Eyes’ test (RMET) [41] is a test of mentalising (‘theory of mind’), the ability to attribute mental states (beliefs, desires) and emotions to other agents. Participants are shown 36 pairs of eyes and must identify the mental state (e.g. playful, frightened, regretful) in each depiction. The test has good internal consistency and test-retest reliability [60, 61].

Current (i.e. state) depression and anxiety were measured by the Beck Depression Inventory (BDI) [62] and the Beck Anxiety Inventory (BAI) [63] respectively. Both tests are used clinically and possess strong psychometric validity and reliability [64–66]. The BDI reflects depressive symptoms over the last fortnight; the BAI reflects symptoms of anxiety over the last month.

### The Non-Suicidal Self-Injury Assessment Tool (NSSI-AT): coding and categorisation of participants

Developed by Whitlock et al. [2], this comprehensive instrument documents the nature and bodily location of any self-injurious behaviours; their functional utility; their recency, frequency and likelihood of reoccurrence; the age of onset of self-injury; the severity of injuries (based on whether these did or should have received medical attention); the social and habitual routines or context around self-injurious behaviours (if, for example, individuals always make sure they are alone); the degree to which participants are habituated to the occurrence of self-injurious behaviour; and whether individuals have sought therapy, their experiences in therapy and their experiences of telling others about their self-injury.

The NSSI-AT lends itself to in-depth qualitative exploration but several aspects of the scale were of especial interest in this analysis, and so we quantified them for comparison between participants. Whilst the scale allowed us to differentiate between participants who had and those who had never engaged in NSSI, we further categorised our self-harming group as follows: current self-harmers were those who had last engaged in NSSI between 1 week and 1 year ago, and who rated themselves as ‘very’ (4 points) or ‘somewhat likely’ (3 points) ‘unsure’ (2) or left the question blank as to whether they would harm themselves again; historic self-harmers were those who had last engaged in NSSI more than 1 year ago and classed themselves as ‘unsure’ (2 points), ‘somewhat’ (1 point) or ‘very unlikely’ (0 points) or left the question blank as to whether they were likely to harm themselves again. This categorisation system allowed us to easily categorise all but two participants who had engaged in NSSI more than 1 year ago but suggested they were ‘very’ or ‘somewhat likely’ to do it again; since NSSI was still a likely option in their behavioural repertoire, these individuals were classed as a current self-harmers.

Of descriptive interest were many of the variables explored in Maddox et al.: for example, the commonest types and bodily locations of NSSI, the typical age of onset of NSSI and the initial motivation for starting, the functional role of NSSI, the extent to which self-injury troubled participants and the ways in which it did so, and participants overall positive and negative thoughts about their experiences around NSSI.

Particular attention was paid to the functional role of self-injurious behaviours. Self-injurious behaviours fulfilled one or more of five roles, example items of each which can be seen in Table 1:

**Table 1:** Functional roles of NSSI

Functional role of NSSI	Example answers to the question ‘I hurt myself …’
Affective imbalance-low pressure (4 items)	‘… to feel something.’ ‘… to change my emotional pain into something physical.’
Affective imbalance-high pressure (3 items)	‘… to relieve stress or pressure.’ ‘… to deal with frustration.’
Social communication and expression (3 items)	‘… in hopes that someone would notice that something is wrong or that so others will pay attention to me.’ ‘… to shock or hurt someone.’
Self-retribution and deterrence (4 items)	‘… as a self-punishment or to atone for sins.’ ‘… so I do not hurt myself in other ways.’
Sensation seeking (4 items)	‘… because I get the urge and cannot stop it.’ ‘… to get a rush or surge of energy.’

Example items for the five functional roles of self-injurious behaviour outlined in the Non-Suicidal Self-Injury Assessment Tool (NSSI-AT)

As in Maddox et al. [15], participants who indicated that they engaged in NSSI only as a means of practicing or attempting suicide were excluded from analysis, though participants who included this as one reason alongside having engaged in NSSI for other reasons were included. As in Whitlock et al. and Maddox et al., responses of ‘strongly’ and ‘somewhat agree’ were collapsed to indicate affirmation of that functional role, whilst responses of ‘strongly’ or ‘somewhat disagree’ were collapsed to indicate denial of that role. We allocated each affirmation within a category a score of 1, and took an average of the number of statements endorsed in each category across participants for a descriptive analysis, but also included scores in each of the five categories as outcome measures in a regression to examine their relationship with the variables reported above.

Indeed, included in our analysis as continuous outcome variables were not only scores in each of these five functional categories, but also

- The range of NSSI behaviours (quantified by giving a score of 1 for each type of NSSI engaged in, such that higher scores indicated that participants engaged in a wider range of NSSI than individuals who consistently used one or two methods)
- The number of bodily locations targeted (quantified by giving each location a score of 1, with higher scores indicating that participants targeted more areas of their body for NSSI)
- The lifetime incidence of NSSI (quantified by giving a score of 1 for up to five occurrences, a score of 2 for 6–20 incidents, a score of 3 for 21–50 incidents and a score of 4 for more than 50)
- The frequency of NSSI in the participant’s most active period of engaging with this behaviour (quantified as follows: a score of 1 if participants engaged in NSSI once in a period of 1, 2 or more years, a score of 2 if they engaged in NSSI once every few months, a score of 3 if they engaged between once a week and 1–3 times a month and a score of 4 if they engaged in NSSI every day or 2–3 times per week).

### Analysis

Following our descriptive report of the type and bodily location of NSSI, initial motivation, age of onset, functional reasons, extent and type of repercussions caused by NSSI and lasting feelings about NSSI, we conducted a number of regression analyses with categorical or continuous outcome measures. Firstly, multinomial regression was used to examine which variables could, individually, correctly categorise participants as current, historic or non-self-harmers: these variables included autistic traits (AQ), depression (BDI) and anxiety (BAI) scores, mentalising score, alexithymia score and sensory profile as reflected in scores for low registration, sensation seeking, sensory sensitivity and sensory avoidance. (We included measures of anxiety and depression in this analysis to specifically test the assertion that autistic self-harmers and non-self-harmers do not differ significantly in depression [15], but results must be interpreted with caution as these measures reflect current psychological health within the last fortnight and month respectively. As such, BDI and BAI scores were not used in the remainder of the analysis below). Regression models tested whether current self-harmers could be differentiated from non-self-harmers, and whether historic self-harmers could be differentiated from non-self-harmers. We therefore included, for each variable that was seen to be a significant predictor of group categorisation, a planned *t* test comparing scores between current and historic self-harmers.

Secondly, stepwise linear regression including only current and historic self-harmers was used to examine the predictive power of autistic traits (AQ), mentalising score, alexithymia score, sensory low registration, sensation seeking, sensory sensitivity and sensory avoidance on four continuous measures: the range of NSSI behaviours, the range of bodily locations targeted, the lifetime incidence of NSSI and the frequency of NSSI in the participant’s most active period of engaging with the behaviour.

In consideration of the five functional roles of NSSI as outcome measures in regression, we took a theory-driven approach in order to reduce the number of statistical tests conducted. Alexithymia was hypothesised to be associated with participants’ use of NSSI to address affective imbalances of both the high pressure and low pressure type. Use of NSSI as a means of expressing and communicating with others was hypothesised to be predicted not only by alexithymia (which relates to one’s ability to verbally communicate emotional states) but by ability to understand other people (mentalising): deficits would theoretically impair communication with others. Sensation seeking as a reason for NSSI was hypothesised to be predicted by the sensation seeking and low registration scales of the Sensory Profile. We did not have hypotheses regarding the prediction of NSSI for the purpose of self-punishment and deterrence, so entered autistic traits, mentalising score, alexithymia score and sensory profile in low registration, sensation seeking, sensory sensitivity and sensory avoidance into a stepwise linear regression.

We conducted a thematic analysis of two open items from the Non-Suicidal Self-Injury Assessment Tool (NSSI-AT). As we took the two questions directly from the questionnaire and these were loaded with prior assumptions (e.g. that something *had* helped reduce or prevent self-injury), our analysis could not be described as fully inductive in nature, despite our attempts to approach it without expectation as to *what* participants would answer to the two items. The analysis was conducted independently by two of the researchers, RLM and NJG, who pursued a thematic analysis in the style of Braun and Clarke [67]. In this conceptualization, the themes in the data do not exist there objectively but ‘reside in our heads from our thinking about our data and creating links as we understand them’ ([68], pp. 205–6). RLM’s familiarity with the quantitative data of the present study, alongside previous literature on self-injury in ASC, was expected to undoubtedly interact and influence her interpretation of the qualitative data. NJG, in contrast, performed her qualitative analysis blind to the quantitative data generated by the same participants and without familiarity with the literature on self-injury in autistic and typically developing populations. The two authors independently followed the pipeline set forward by Braun and Clarke: first, extensively familiarising themselves with the dataset as a whole, then generating initial codes for the data, with some quotations from participants fitting into multiple categories; thirdly, identifying latent themes across the codes. At this point, the authors came together to review the themes that they had identified independently. In multiple meetings over the course of several weeks, they discussed and revised their initial mind-maps and thematic tables, until they had unanimously defined and named the final themes that appear in this analysis.

## Results

### Group demographic information

The demographic details of each group are given in Table 2:

**Table 2:** Demographic details for each group

	Percentage female	Age (years)	Age at diagnosis (years)	Percentage employed	Percentage qualified to at least degree level	Percentage with comorbid psychiatric diagnoses	Percentage taking medication
Current self-harmers (*n* = 49)	75.5% (*n* = 37)	41.2 (3.4)	33.4 (13.8)	53% (*n* = 26)	66% (*n* = 21)	85.7% (*n* = 42)	65.3% (*n* = 32)
Historic self-harmers (*n* = 27)	63% (*n* = 17)	43.5 (15.8)	36.1 (17.1)	37% (*n* = 10)	63% (*n* = 17)	81.5% (*n* = 22)	37.1% (*n* = 10)
Non-self-harmers (*n* = 27)	59.2% (*n* = 16)	43.0 (13.6)	34.2 (16.2)	66.6% (*n* = 18)	70.4% (*n* = 19)	48.1% (*n* = 13)	33.3% (*n* = 9)

Average age and age at diagnosis for each group (standard deviations in brackets). Also included are percentages of female participants; participants who were employed, qualified to at least degree level, suffering from a comorbid psychiatric diagnosis, and taking medication

Participant groups did not differ with respect to current age (*F* [2, 102] = 1.077, *p* = .345) or age at diagnosis (*F* [1, 102] = 1.877, *p* = .158). Neither was the distribution of female participants across groups significantly different (χ^2^(2) = 1.906, *p* = .086) nor was the distribution of participants who were employed (χ^2^(2) = 4.341, *p* = .114), or those who had a degree (χ^2^(2) = .350, *p* = .840). However, the distribution of participants who were taking medication (χ^2^(2) = 7.720, *p* = .021) and the distribution of participants with additional psychiatric comorbidities (χ^2^(2) = 12.814, *p* = .002) were significantly different: the participants in both self-harming groups were more likely to be experiencing psychiatric comorbidities, and the current self-harming group were more likely to be taking medication at the time of the study. Group scores in each of the variables of interest are displayed in Table 3.

**Table 3:** Group scores in experimental variables

	Depression (BDI)	Anxiety (BAI)	Alexithymia (TAS-20)	Autism spectrum quotient (AQ)	Number of correct answers: RMET	Sensory low registration	Sensory seeking	Sensory sensitivity	Sensory avoidant
Current self-harmers (*n* = 49)	25.6 (13)	24.6 (11)	65.9 (11)	39.1 (9)	23.6 (8)	43.1 (10)	35.9 (9)	52.9 (10)	53.0 (11)
Historic self-harmers (*n* = 27)	22.9 (15)	22.3 (14)	60.4 (12)	37.8 (8)	24 (6)	39.9 (10)	37.4 (8)	47.6 (13)	47.2 (12)
Non self-harmers (*n* = 27)	14.6 (10)	13.1 (9)	56 (15)	35.6 (10)	23.3 (9)	38.8 (14)	38.7 (12)	44.1 (14)	46.2 (17)

Average scores for each group on each variable of interest (standard deviation in brackets)

BDI and BAI scores, on average, fell in the range of mild depression (10–19) and mild anxiety (8–15) for non-self-harmers and moderate to severe depression (20–30) and moderate anxiety (16–25) for current and historic self-harmers [64, 69]. Sixty-three of 103 participants (61%) scored above the 61 cut-off point for clinical levels of alexithymia [50]: the lowest score was 33, and 9 participants fell just below the cut-off by scoring 59 or 60.

On the AQ, all but 6 participants scored ≥ 26, a cut-off which correctly categorises 83% of autistic individuals [70]; on average, autistic individuals tend to receive scores of approximately 35 [56], consistent with our groups. Likewise, scores in the RMET are similar to those seen in other publications with autistic participants [41, 71].

Group averages in the subscales of the Sensory Profile suggested that participants scored above test norms (based on nearly 500 typically developing individuals) in low registration, sensory sensitivity and sensory avoiding. All groups scored below test norms for sensation seeking.

### Descriptive analysis of self-injury

Of the 76 current and historic self-harmers, 60 could recall the onset of self-injury at an average age of 15.1 years (SD = 10.8). Seven others also estimated that they began self-harming in childhood or early adolescence. The reason they first engaged in self-injury, the range and bodily location of NSSI are summarised in Table 4.

**Table 4:** Range and bodily location of NSSI in autistic individuals

Type of NSSI	Percentage (%) of participants
Severely scratched or pinched with fingernails or other objects to the point that bleeding occurs or marks remain on the skin	72.4% (*n* = 55)
Cut wrists, arms, legs, torso or other areas of the body	50% (*n* = 38)
Banged or punched objects to the point of bruising or bleeding	44.6% (*n* = 33)
Punched or banged yourself to the point of bruising or bleeding	44.6% (*n* = 33)
Bitten yourself to the point that bleeding occurs or marks remain on the skin	41.2% (*n* = 31)
Intentionally prevented wounds from healing	38.2% (*n* = 29)
Ripped or torn skin	34.2% (*n* = 25)
Burned wrists, hands, arms, legs, torso or other areas of the body	30.1% (*n* = 22)
Rubbed glass into skin or stuck sharp objects such as needles, pins and staples into or underneath the skin (not including tattooing, body piercing or needles used for medication use)	27.6% (*n* = 21)
Carved words or symbols into the skin	20.5% (*n* = 15)
Engaged in fighting or other aggressive activities with the intention of getting hurt	11% (*n* = 8)
Tried to break your own bones	8.2% (*n* = 6)
Ingested a caustic substance(s) or sharp object(s) (bleach, other cleaning substances, pins, etc.)	6.8% (*n* = 5)
Banging head against walls, hard surfaces	6.8% (*n* = 5)
Broke your own bones	2.7% (*n* = 2)
Dripped acid onto skin	2.7% (*n* = 2)
Pulled out hair, eyelashes or eyebrows (with the intention of hurting yourself)	1.7% (*n* = 1)
Other (avoided taking medication or seeking healthcare as a form of self-harm; tried to choke/strangle myself; took small overdoses of paracetamol or paracetamol; poured boiling water over hands; provoking an animal to bite; trying to get hit by traffic; tried to set myself alight; dropped heavy objects onto myself; tried dropping off heights; masturbated with metal objects that caused me to bleed)	24.7% (*n* = 18)
Bodily site of NSSI	
Arms	61.8% (*n* = 47)
Hands	58.1% (*n* = 43)
Head	47.4% (*n* = 36)
Wrists	42.5% (*n* = 31)
Face	35.1% (*n* = 26)
Fingers	31.6% (*n* = 24)
Stomach or chest	31.5% (*n* = 23)
Thighs	28.8% (*n* = 21)
Calves or ankles	14.5% (*n* = 11)
Lips or tongue	13.7% (*n* = 10)
Shoulders or neck	11.8% (*n* = 9)
Breasts	9.2% (*n* = 7)
Genitals or rectum	6.6% (*n* = 5)
Feet	2.7% (*n* = 2)
Back	2.7% (*n* = 2)
Eyes	1.4% (*n* = 1)
Initial motivation for NSSI	
I was angry with myself.	38.2% (*n* = 29)
I accidentally discovered it—I had never seen or heard of it before.	38.2% (*n* = 29)
I was upset and decided to try it	30.3% (*n* = 23)
I was angry with someone else.	15.1% (*n* = 11)
It felt good.	15.1% (*n* = 11)
I wanted someone to notice me and/or my injuries.	11% (*n* = 8)
I cannot remember.	8.2% (*n* = 6)
I wanted to shock or hurt someone.	27.7% (*n* = 2)
It seemed to work for other people I know.	27.7% (*n* = 2)
I did it because I had friends who did it and I wanted to fit in.	27.7% (*n* = 2)
I saw it on a movie/television or read about it in a book and decided to try it.	27.7% (*n* = 2)
I read about it on the internet and decided to try it.	1.4% (*n* = 1)
It was part of a dare.	1.4% (*n* = 1)
Other (‘Whilst not remembering the exact first time, I know it was initially an attempt to FEEL my own self-loathing—to be able to grasp and feel the feeling’; ‘It reduced my stress’; ‘I was having what I now know was a meltdown and did it in desperation to “do” something’; ‘I needed to do so something to ease the pain I felt inside’; ‘I hated myself’; ‘It just happened. It was like a compulsion and I could not control myself at all’; ‘I copied my dad’; ‘I wanted to be humiliated, “told you so”’; ‘I was so stressed’; ‘I just wanted out of the situation I was in’; ‘I was frustrated by other people’s talking and noise and rule-breaking and needed something to distract me’; ‘I was depressed’; ‘Boredom’; ‘I was so frustrated, cornered, it felt like the last resort’; ‘I was trying to understand what had happened to me at a doctor’s surgery’; ‘full of self-hatred and confusion’.)	21.1% (*n* = 16)

Participants report the their methods of self-injury, the bodily areas most commonly targeted and initial motivations for starting

**Fig. 1:**
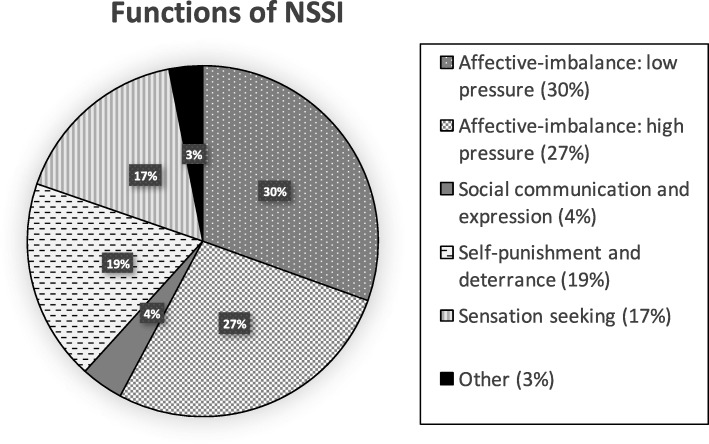
Functions of NSSI. This chart depicts the average number of statements endorsed in each category as a percentage

To analyse the functional role that NSSI plays or played, we took an average of the number of statements endorsed from each of the five categories. The average of each category is displayed in Fig. 1 as a percentage. On average, current and historic self-harming participants endorsed 2.6 statements from the affective-imbalance low pressure category (which has 4 statements overall), making this the most common reason for NSSI. This was followed by affective-imbalance high pressure (2.3 statements on average, out of 3 possible statements), self-punishment and deterrence (1.6 statements on average, out of 4 possible statements), sensation seeking (1.4 statements on average, out of 4 possible statements) and social communication and expression (.33 statements on average, out of 3 possible statements). A small number of additional ‘other’ reasons were offered. Some of these were rewording of items from the other categories (‘frustration’, for example, could be recoded in the affective-imbalance high pressure category; ‘punishment for incompetence’ could be recoded in the self-punishment and deterrence category). Other motivations for NSSI that were less transparent to categorise or indeed separate included ‘to stop sensory input from overwhelming me’; ‘To look ugly, deformed’; ‘I wanted to disappear from an unbearable situation’; ‘To appease voices’; ‘Because it helps me deal with my eating disorder’; ‘Over a relationship’; ‘Distraction’; ‘So I don’t hurt someone else or break something’; ‘Loneliness’; ‘To stop being lost to get out of lost the empty black where I don’t know what to do. It helps me to focus and get out of the black, to have focus to move on’.

**Fig. 2:**
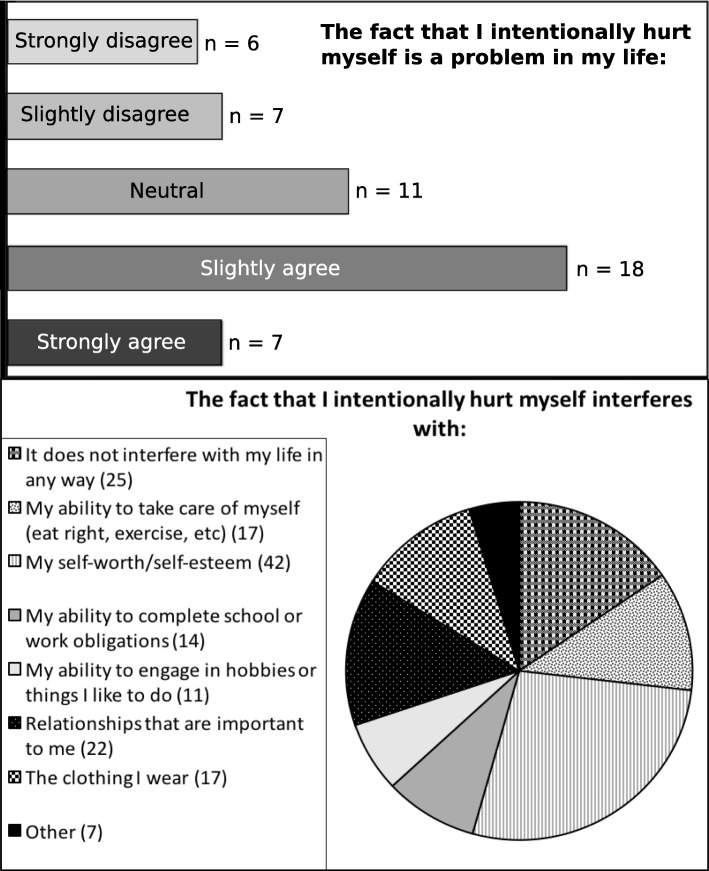
Negative repercussions of NSSI. The chart depicts participant responses to questions on the NSSI-AT as to **a** whether self-injury is a problem in their life and **b** whether self-harm was problematic in relation to seven repercussions specified by the NSSI-AT. Several participants offered additional problems caused by NSSI (‘Other’) which included: ‘It causes anxiety in public places as I don’t want other people to watch me while I am doing it’; ‘Being a mother and role model for my son’; ‘Hiding when it hurts how I move or if touched’; ‘I’m ugly. No man would want me and people won’t want to be my friends as they’d be ashamed to be seen with me’; ‘I don’t know what the long-term effects might be and that sometimes worries me’; ‘Constant trips to A&E’ (hospital); ‘Ashamed of my appearance’

Participant responses to the query as to whether self-injury was a problem in their life, and how so, are displayed in Fig. 2.

Lasting feelings about NSSI, a summary question at the end of the NSSI-AT, corroborated participants’ concern over the physical marks of NSSI. Twenty-eight participants endorsed a statement that their scars are a constant reminder of a bad time in their life; 19 that their scars are a source of embarrassment. Ten people took a more positive view of their scars as their ‘battle wounds’. Seventeen participants reported that they found it hard to think about or talk about their experience with NSSI, but surprisingly, the most commonly endorsed feeling (30 participants) was that their NSSI had not impacted on their life much at all. Nineteen participants thought that they had learnt something from their experience with NSSI and had grown emotionally/mentally; 5 that they could now help others who self-injure; 2 reported that NSSI had caused anxiety in their relationships, but 7 reported that talking about their experience with NSSI had brought them closer to people they care about.

### Statistical analysis: predictors of NSSI

Variables which predicted the categorisation of participants as current, historic or non-self-harmers were alexithymia (χ^2^(2) = 10.677, *p* = .005), BDI scores (χ^2^(2) = 12.313, *p* = .002), BAI scores (χ^2^(2) = 19.299, *p* < .001) and sensory sensitivity (χ^2^(2) = 9.953, *p* = .007). Alexithymia explained 11% (Nagelkerke *R*_2_) of the variance in categorisation, with the model able to differentiate significantly between current and non-self-harmers in accordance with their alexithymia scores (*b* = .062, Exp [*b*] = 1.064, *p* = .002) but not between historic and non-self-harmers (*p* = .232); planned comparison showed that the difference between current and historic self-harmers was only marginal (*t* [74] = 1.968, *p* = .053). Scores on the depression inventory explained 13% of the variance (Nagelkerke *R*^2^), with the model able to correctly differentiate current from non-self-harmers (*b* = .068, Exp [*b*] = 1.070, *p* = .002) and historic from non-self-harmers (*b* = .053, Exp [*b*] = 1.055, *p* = .02), and planned comparison showing no significant difference between current and historic self-harmers. Scores on the anxiety inventory explained 19% of the variance (Nagelkerke *R*^2^), with the model correctly differentiating between current and historic self-harmers (*b* = .107, Exp [*b*] = 1.113, *p* < .001) and between historic and non-self-harmers (*b* = .091, Exp [*b*] = 1.095, *p* = .003), and planned comparisons showing no significant differences between current and historic self-harmers. Sensory sensitivity explained 10% of the variance (Nagelkerke *R*^2^), but the model only significantly differentiated between current and non-self-harmers (*b* = .063, Exp [*b*] = 1.065, *p* = .004); the planned comparison between current and historic self-harmers (*t* [74] = 2.005, *p* = .049) was only marginally significant.

This approach to regression, of course, fails to address the issue of multicollinearity, given that many of these variables would theoretically be expected to correlate with one another. As such, we added all variables (AQ, BDI, BAI, alexithymia, mentalising score and the four sensory variables) into a binomial regression. Together, they significantly predicted categorisation of participants as current, historic or non-self-harmers (χ^2^(2) = 34.73, *p* = .022), and explained 38% of the variance. However, given the correlations (i.e. shared variance) between the variables (Additional file 1: Table S1), no single variable emerged as a significant predictor in the model; greater power would be required to tease out the contributions of each variable.

Stepwise linear regression was conducted to look at several continuous outcome measures, namely the range of self-injurious behaviours, the range of bodily locations targeted, the lifetime incidence of NSSI and the frequency of NSSI in the participant’s most active period of self-injury. None of the variables significantly predicted the range of self-injurious behaviours. The only significant predictor to remain in a model of the range of bodily areas targeted (*F* [1, 62] = 5.157, *p* = .027) was sensory avoidance (*b* [standardised coefficient] = .279, *t* = 2.271, *p* = .027). Likewise, the only predictor to remain in a model of lifetime incidence (*F* [1, 62] = 7.715, *p* = .007) was sensory avoidance (*b* = .335, *t* = 2778, *p* = .007). The only predictor to remain in a model of frequency of behaviours in most active period (*F* [1, 62] = 4.264, *p* = .043) was sensory low registration (*b* = .256, *t* = 2.065, *p* = .043).

As regards engagement in NSSI for the purpose of regulating low-energy states such as depression or dissociation, our hypothesis was not supported: alexithymia was not a significant predictor of participants’ endorsement of statements about NSSI related to regulating these low-energy states. In contrast, the hypothesis that alexithymia would predict use of NSSI to regulate high-energy states was supported (*F* [1, 74] = 5.065, *p* = .027), with participants high in alexithymia more likely to endorse statements about engaging in NSSI for the purpose of regulating high-energy states. In a stepwise regression predicting use of NSSI for communicative purposes (*F* [1, 74] = 5.065, *p* = .027), alexithymia (*b* = .255, *t* = 2.251, *p* = .027) was a significant predictor but mentalising performance was not. Neither sensory low registration nor sensory seeking predicted NSSI for the purpose of sensory seeking. None of the variables predicted NSSI for the purpose of self-punishment and deterrence.

### Qualitative analysis: autistic voices on self-injury

**Fig. 3:**
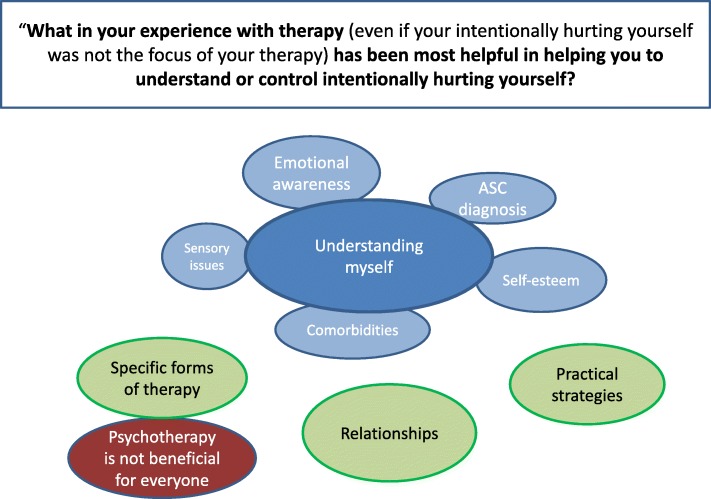
First thematic map. Figure depicts themes and subthemes around experiences with therapy

We chose to analyse two qualitative items from the NSSI-AT. The first item was: ‘What in your experience with therapy (even if your intentionally hurting yourself was not the focus of your therapy) has been most helpful in helping you to understand or control intentionally hurting yourself?’. A thematic map for this question is depicted in Fig. 3:

Sixty-three of our 76 participants responded to this question, and 4 core categories were identified within the data. The most overarching of these was *understanding myself*, which encompassed the themes of developing *emotional awareness*, understanding the roles of *self*-*esteem* and *sensory issues*, getting and understanding an *ASC diagnosis* as well as acknowledging the role of *comorbidities*. In addition, three further themes were found: *Specific forms of therapy* which helped, therapeutic and personal *relationships* were important and the *practical strategies* that therapy focussed on developing and which helped manage or reduce NSSI. Finally, a contrasting theme was found, that *psychotherapy was not beneficial for everyone*. Please see Additional file 1: Table S2 for a thematic table of responses.

#### Understanding myself

The way that therapy had helped participants to better understand themselves and their reasons for NSSI was central to the data*.* This understanding described in this theme took the form of five sub-themes.

*Emotional awareness*: Participants spoke most widely about how emotional experiences were at the root of their self-injury and that understanding what these were, what caused them and then learning strategies to manage those emotions, often helped reduce their NSSI. As this was such an important but diverse subtheme, we sub-divided it into five aspects of emotional awareness.

i. Understanding the cause of emotions (e.g. ‘… starting to understand my emotions and what is “upsetting”’—P75).
ii. Identifying emotions: some participants highlighted a difficulty in identifying emotions which could exacerbate NSSI *(*‘[Understanding] that I hurt myself out of [...] fear of showing confusing emotions’—P74) and alleviate NSSI when assisted (‘Learning to name my thoughts and feelings [...helped with my NSSI]’—P11)*.*
iii. Expressing emotions: participants mentioned how learning to articulate their feelings to others was something that had helped with NSSI (‘Reading and speaking to other people openly’—P72). The process of articulating emotions also helped one participant to understand their own feelings more fully (‘Verbalising some feelings so that I can understand them better’—P17).
iv. Emotions in control: there was a sense from several participants that their emotions controlled them or were something which needed to be controlled. Increasing emotional awareness and learning strategies to manage emotions changed that dynamic so that participants were more in control, and seemed to consequently help reduce NSSI (‘Not letting them [thoughts and feelings] take control over me’—P11).
v. Management strategies: once identified, it was possible for participants to put strategies in place to alter, manage or control these difficult emotions (‘Learning that I have other ways to change how I feel - or don’t feel - and learning to do those things.’—P22). The methods of managing difficult emotions which helped participants were stress-reduction techniques, such as MBSR and relaxation techniques, hypnotherapy, having regular times to check in with emotions. However, despite many strategies helping participants to ‘stop letting [their] emotional pain build up’ (P64) and thus manage their NSSI, one participant eloquently described how management strategies were in fact less helpful than seemingly more basic emotional awareness work (‘I haven’t attended therapy for over a decade, but better understanding my anger and the causes of it (not necessarily any relevant, suggested coping mechanisms/strategies) was the most constructive in regards to self-injury’—P12).

Other subthemes of ‘Understanding myself’ were:

*Sensory issues:* two participants acknowledged sensory stimulation can be tied up with their need to engage in NSSI, and that increasing awareness of physiological states (‘checking in with myself every few hours to know if I am hungry/too hot/too cold/thirsty/tired’—P39) and being aware of when they needed to do something to change that was helpful.

*Self-esteem:* several participants described how through therapy, they had become aware of the role of low self-confidence or self-esteem in their NSSI, such that building their self-esteem made NSSI less likely (‘That I hurt myself out of low self-love and low self-esteem (which did improve in time, and so did self-injuries become less possible’—P74).

*Getting a diagnosis of ASC: *being diagnosed with ASC was cited by several participants as beneficial to understanding and/or reducing their NSSI (‘Actually having my ASD diagnosis has been the most helpful thing’—P10). Notably, the five participants who made statements in this vein were diagnosed after the age of 30, two in their sixties.

*Comorbidities:* three participants also acknowledged the importance of addressing and treating their comorbidities in understanding and controlling their NSSI, naming depression, anxiety, OCD and eating disorders (P71, P13, P58).

As a theme, ‘Understanding myself’ seemed the key aspect of therapy which helped participants control and understanding their NSSI. This psychotherapeutic work appeared to enable participants to gain insight into their own feelings, behaviours and other difficulties and to reflect on these in a less judgmental, accepting way. It is possible that this more compassionate attitude to the self made NSSI less of viable option.

#### Practical strategies

As well as the psychotherapeutic work which was central to most participants’ experience of therapy, the next theme described learning practical strategies (the next theme) to cope with urges to self-injure, with examples given such as wearing false nails, elastic bands or drawing in red. There is a link, here, with the emotional awareness and the sensory issues subthemes of ‘Understanding myself’, as these strategies were described for the purpose of managing emotions and sensory experiences once these were identified. For example, finding an alternative sensory stimulation to NSSI was found to be helpful for one participant (‘We did however explore options of generating strong sensory input without causing injury (similar to ‘skills lists’ for BPD)’—P5). Although not aimed at eliminating NSSI itself, one participant (P44) explained how they would reduce alcohol consumption if they intended to self-harm, a sort of damage limitation strategy.

#### Specific forms of therapy

In this theme, participants mentioned specific forms of therapy, in addition to the relaxation techniques mentioned above, which were useful. These included occupational therapy (P32), CAT (P10) and CBT (P48); conversely, one participant expressed that CBT had made them feel much worse (P10).

#### Relationships

Several participants mentioned how the therapeutic relationship was itself beneficial, simply for having ‘regular time’ to ‘check in’ (P27), a safe place to talk and the feeling of ‘being heard’ (P48) by ‘someone who understood’ (P62). Similarly, three participants acknowledged the role of social connections not just within the therapeutic setting in reducing their NSSI (‘Therapy was helpful but when alone for periods in my life, I will regress to self-harming’—P8).

#### Psychotherapy is not always beneficial

This theme, which stands in contrast to the rest of the responses to this question, importantly reflects assertions that therapy undertaken had been at best unhelpful and at worst detrimental. Some participants expressed their confusion and difficulty knowing how to respond to questions from therapists *(*‘Therapy not understand autistic person they use their understanding of how they operate to judge an autistic operating system so all it does is give confusion they get cross and I feel sad and lost because I am not being good and compliant’—P23). Another participant described the group therapy they had received as ‘totally inappropriate’ and ‘traumatising’ (P46). There were many comments that therapy had been ineffective but where participants did not elaborate on why (P14, P28, P38, P41, P47, P55). It is possible that a ‘one size fits all’ approach to psychotherapy may have also been the reason that these participants found no value in the therapy they had been offered.

**Fig. 4:**
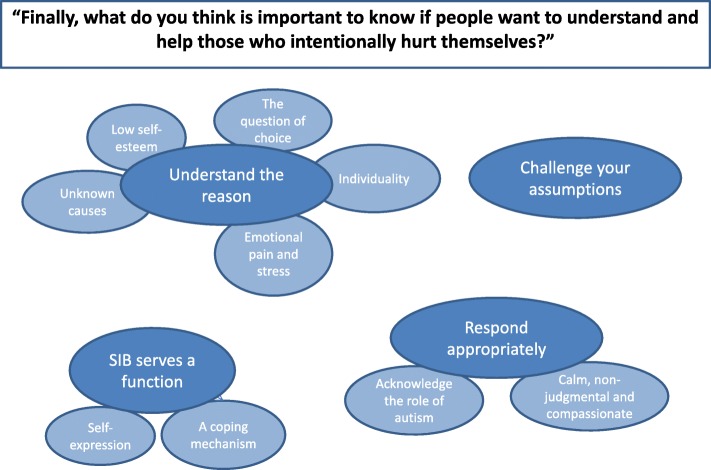
Second thematic map. Figure depicts themes and subthemes exploring the themes and subthemes around messages for people who want to understand and help self-harmers

The second set of responses we analysed were in response to the following question: ‘Finally, what do you think is important to know if people want to understand and help those who intentionally hurt themselves?’**.** A thematic map for this question can be seen in Fig. 4.

Four themes emerged from analysis of this item. It was clear from the data that participants wanted others to *understand the reasons* behind NSSI. Instead of resorting to stereotypes about people who self-injure, participants were definite that others should *challenge their assumptions* and seek to look beyond the behaviour itself to the underlying reasons for it. The most prominent reason, and a theme in its own right, was that *NSSI served a function* for most participants as a coping mechanism or a means of self-expression. With this new knowledge, others involved with those who self-injure should *respond appropriately*, that is calmly, non-judgmentally and compassionately, whilst acknowledging the unique role of autism in these individuals who self-injure. A full table of themes and quotes can be seen in Additional file 1: Table S3.

#### Understand the reason

Participants wanted others to understand that people engage in NSSI for a reason and that people should seek to *understand the reason* in order to better understand NSSI and help self-harmers. It was important to participants that people realise that NSSI in itself was not necessarily the problem (or indeed *a* problem), but rather ‘a symptom of a significant problem’ (P41), and that ‘the outer wound only hints at a much more painful inner (hidden) wound’ (P59).

Participants wanted others to know that the main reasons behind the use of NSSI were that it was a response to *low self*-*esteem*, *emotional pain*. It was acknowledged that that there is great *individuality* in the motivations to self-injure, and that sometimes there are *unknown reasons* behind participants’ NSSI. NSSI may or may not be a *choice*. We explore each subtheme in turn.

*Emotional pain*: The overwhelming majority of participants cited difficult emotional experiences as prompting their self-injury. Specifically, participants mentioned confusing emotions, anger, stress, anxiety, frustration, ‘pressure’, ‘emotional pain’, ‘hurting inside’ and stress as the precursors to self-injury, and one participant suggested that NSSI serves as ‘a coping mechanism to convert emotional pain into physical pain’ (P9).

*Low self-esteem*: One of the less frequent causes mentioned by participants was low self-esteem, which was cited by one participant as resulting from the difficulties they faced as a person with autism *(*‘I hurt myself because of my self-hatred and desire to punish myself for the problems I have with everything due to my Aspergers’ (P47).

*Unknown causes*: Whilst theoretically self-injury should always serve a functional purpose, several participants explained that people who engage in NSSI may not fully understand why they do it themselves (‘I really don’t know why I did it’—P17)*.*

*Individuality*: Participants expressed the importance of recognising the individuality of the self-injurer before making assumptions about why someone is turning to NSSI *(*‘It’s really important to find out how to address each individual, there is a common misconception that we all fit in the same box … we really don’t’—P70).

*The question of choice*: This subtheme reflected an interesting dichotomy in the data. A number of participants (P6, P17, P72, P26, P65, P70, P45, P46) expressed a lack of conscious control or choice over self-injury (‘That it happened just like that; I had no control over hurting myself’—P6) and a clear aversion to the behaviour (‘They don’t want to do it’—P44). The word ‘compulsion’ was used twice (P26, P65), the word ‘addiction’ twice (P72, P46). However, the way NSSI was framed by other participants (P5, P7, P12, P16, P18, P21, P27, P39, P41, P46) suggested it was a conscious choice, a strategy that people could choose to use (‘I have no problem with intentional hurting. I know when and why … I can either not do the act [the stressful thing] or cause some pain to achieve homeostatic balance’—P7)*.* In line with this, some explained that NSSI should not always be seen as a negative thing (‘That sometimes, if controlled appropriately, it can be a helpful way to control overwhelming feelings. As long as it is controlled and isn’t causing huge degrees of harm, then there could be many worse things the person could be doing’—P16). The words ‘choice’ or ‘choose’ were used by two participants (P7, P46), whereas others use the word ‘strategy’ (P21) or ‘outlet … a form of expression’ (P12), and mention their ability to ‘control’ or ‘resist’ it (P16, P39). Interestingly, one participant used the word ‘compulsive’ to distinguish their self-injury from ‘impulsive’ acts (P27). This participant appeared to be using the word in a different sense to those described above, who seemed to use ‘impulse’ and ‘compulsion’ in an interchangeable way that suggested they were ‘forced to do it’ (e.g. P25, P26). Participant 27’s use of the words seems to correspond to their more scientific definitions, ‘impulsive’ as ‘a predisposition toward rapid, unplanned reactions to internal or external stimuli with diminished regard to the negative consequences of these reactions’, and ‘compulsive’ as ‘a tendency to perform unpleasantly repetitive acts in a habitual or stereotyped manner to prevent perceived negative consequences’ ([72], pp. 591). The words used by some participants suggested that they might feel both elements of control and elements of helplessness: one (P46) spoke of self-injury first as a ‘choice’ and then as ‘an addiction’ (implying automaticity and lack of volition) in the same sentence.

#### Challenge your assumptions

Several participants believed there are public misperceptions about those who self-injure and that these should be challenged. The most commonly cited perceptions that participants thought were prevalent were that those who self-injure are ‘crazy’ (P9), ‘attention seekers’ (P50), ‘drama queens’ (P18), ‘irresponsible and over emotional’ (P18), or had a personality disorder (P50). Another participant alluded specifically to inaccurate attitudes of some health professionals regarding the motivation for NSSI (‘Not everyone who self-injures does it for attention. When medical personnel are treating these people, they deserve the same respect and treatment that anyone else would get. Don’t assume they ‘like pain’ and refuse them anaesthetic’—P22). Notably, with a link to the choice subtheme above, some participants found it unhelpful that self-injury was automatically seen as a negative thing (P5, P41).

#### NSSI serves a function

Participants wanted others to know that NSSI has a functional role in managing a range of difficulties. This links to the choice subtheme discussed above, where some participants expressed that NSSI was not always a negative thing—that it was not, in itself, a distressing thing, and was something they would struggle to do without (‘If anyone had tried to get me to stop, I would have been much, much worse’—P27). The first of two subthemes of this theme was the idea of NSSI as a ‘coping mechanism’ (P9), a tool participants could use to ‘self-regulate or cope with overwhelming emotions or find brief relief from suffering’ (P28), such that they could continue their day and participate in activities like work and social interactions that they would have otherwise been unable to complete (‘Some days things happen and I feel so overwhelmed that I feel like I will break completely and I have no idea how I can get through my day (I work). Seeing the blood is like flipping a switch … then I can go back and get on with meetings and talking to people’—P3). The second subtheme was the idea of NSSI as means of *self*-*expression*, ‘much like any creative or artistic outlet … a form of expression that some people turn to when words or other communicative methods do not fully convey how they feel’ (P12)—a method which served a function when participants had no alternative means of expressing themselves.

#### Respond appropriately

Finally, having helped others to understand the causes behind NSSI, people should use this knowledge to respond appropriately to the self-injurer in a way that will support them best: *calmly*, *non*-*judgementally and compassionately*, and recognising the *autism*-*specific needs* of the individual.

*Calmly, non-judgmentally and compassionately*: A calm, non-judgmental and compassionate approach was mentioned as an appropriate way to respond (‘Never get emotional about it with someone—P10; ‘Be patient and understanding. Non-judgemental and considerate’—P49). Above all, participants expressed that others should make the person who self-injures aware that they are not alone, that they are loved, supported and cared for (‘Be there for them. Make sure they know they’re loved. DON’T leave them alone’—P53; ‘People need relationships, love and appreciation’*—*P56)*.*

*Acknowledge the role of autism*: Participants explained that others should acknowledge the role that autism has in NSSI, especially when trying to discuss it. They should potentially modulate their communication appropriately *(*‘You are speaking a different understanding and it is so hard to find a moment where understanding touches’*—*P23; ‘To not beat around the bush. Just try and speak openly about it’—P24). One participant conceptualised their NSSI as part of their autism, highlighting that NSSI may need to be conceptualised differently in autistic and non-autistic people (‘It’s a part of my autism - a repetitive, ritualistic, stereotyped behaviour that has developed with me for the past 20 years’—P27).

## Discussion

The prediction and characterisation of self-injury in autistic people is of high clinical importance, given the relationship between self-injury and later suicidality [3–9]. Our mixed-methods approach addressed the dearth of research on self-injury in autistic people without intellectual disability by validating and extending findings from a previous investigation of self-injury in this population [15]. The features of self-injury were remarkably similar in this considerably larger dataset of autistic individuals. An adolescent age of onset of NSSI was confirmed (12.7 years in Maddox et al., 15.1 years in the present study), which mirrors the typical age of NSSI onset in neurotypical participants [1, 73]. As in Maddox, descriptive analysis showed that our participants most commonly engaged in scratching, pinching or cutting, and most often targeted the hands and arms; self-anger and upset were common initial motivators, but the largest proportion of our participants claimed to have stumbled on self-injury ‘accidentally’ without having seen or heard of it from external sources. Whilst Maddox et al. found no difference in the prevalence of self-injury in a direct comparison of autistic adults diagnosed in childhood and those diagnosed after 18, we likewise found no significant differences in age of diagnosis between current, historic and non-self-harming autistic adults, suggesting that no group is of particular risk. Our participants did differ from those in the previous study in respect of biological sex, with Maddox and colleagues reporting a greater number of autistic women than men in their self-harming group. Respondent bias might possibly have influenced these findings in both cases: most participants in the previous study were male (24 out of 42), whereas the majority of our participants (70 out of 103) were female. Sex differences in autistic self-harmers are of interest given that NSSI tends to be more prevalent in women generally, with the ratio of women to men especially greater in clinical populations [74].^3^ Our data suggests that this assumption cannot be extended to autism: self-injury, in ASC, should be of concern to clinicians of male *and* female patients.

An important goal highlighted in this study is the need to identify autistic individuals at heightened risk for self-injury, and we consequently aimed to extend the previous work in this area with consideration of the variables that might be of clinical importance in predicting the presence of self-injury.

### Predictors of self-injury: alexithymia, depression, anxiety and sensory differences

Alexithymia, difficulty identifying one’s own emotional states, was a prime candidate of interest given its common presence in clinical populations who self-injure [16–20]. This construct is not entirely equivalent to emotional dysregulation (operationalised by Maddox et al. with the Emotion Regulation Scale [75]), but encapsulates the latter in combination with difficulty identifying and understanding one’s emotions [76, 77]. With this substantially larger sample, alexithymia was a significant predictor of self-injury, with current self-harmers exhibiting the highest levels of alexithymia followed by historic and then non-self-harmers. Our analysis further connected alexithymia to NSSI for the functional purpose of regulating high-energy states and as a means of social influence (communication and expression). This corroborates our theory-driven interest in this variable: individuals with alexithymia have difficulty identifying, and indeed regulating, high-energy states such as anger, agitation, frustration and anxiety, and by its nature alexithymia describes a difficulty in *expressing* emotional states that would make communication difficult. The importance of this variable also emerged in qualitative responses, where participants spoke about how learning to identify and express emotions, and understand the cause of emotions, was helpful. The use of NSSI to ‘control overwhelming feelings’, and the ‘traumatic’ and ‘frustrating’ difficulty of communicating how they feel, also corroborated our quantitative data around alexithymia.

A growing literature has highlighted the importance of diagnosing comorbid alexithymia, which may explain some of the socioemotional and communicative deficits of autism and be worthy of targeted intervention [78–83]. Our analysis, indeed, suggests that alexithymia may be of clinical relevance in identifying those at particular risk of self-injurious behaviour. The decreased levels of alexithymia in the historic as compared to current self-harmers does, however, create an interesting puzzle for future research. Alexithymia is generally conceptualised as a stable construct (a trait) [84–86], though the temporal stability of the factors (difficulty identifying feelings, difficulty describing feelings and externally orientated thinking) has been seen to differ, the former two to increase when patients are in depressive episodes [87]. Current self-harmers were indeed marginally more depressed than historic self-harmers, but not significantly so.

Difficulty identifying and communicating one’s own emotional states (alexithymia) is closely related to the ability to identify and understand the emotional and mental states of *other* people. Impairments in this latter ability, commonly known as ‘theory of mind’ or ‘mentalising’, were, like alexithymia, hypothesised to put participants at particular risk of self-injury. Interestingly, this hypothesis was not borne out: deficits in the popular ‘Reading the Mind in the Eyes’ task (RMET) [41] were unrelated to the presence of self-injury, its range or frequency or its use for the functional purpose of communicating with others. Notably, previous literature has failed to link NSSI to mentalising deficits, although these are common in clinical populations notorious for self-injury [31–33]. A tenuous link has been made between the two with therapeutic attempts to alleviate self-injury through improving mentalising ability [36–38]. Problematically, the described therapeutic approach conflates strengthening mentalising ability with alleviating alexithymia, involving as it does increasing awareness and identification of emotions and communicating about them. Importantly, mentalising and alexithymia are distinct constructs, and there is debate as to whether the RMET indexes mentalising (the common assumption and the assertion of the authors, who validated it against other mentalising tasks [88–90]) or recognition of emotional expressions [91], deficits in which are associated with alexithymia. Adopting the latter view leaves open the question as to whether mentalising deficits really do increase the risk of self-injury, but the implication from our data, given the relationship between NSSI and TAS-20 scores but not between NSSI and RMET, is that the inability to identify and express one’s emotions is of greater concern for self-injury than is the ability to recognise emotions in others, even if the latter would theoretically impair communication.

Further variables of clinical interest for the risk of self-injury were depression and anxiety. Depression was examined by Maddox et al., who found no difference between their self-harming and non-self-harming autistic groups. We suggest the likely reason for this lack of difference was their indiscriminate categorisation of self-harming participants, a group containing current self-harmers and those we would classify as ‘historic’.^4^ Indeed, where current depression significantly predicted the dichotomous likelihood that participants had ever engaged in NSSI, planned *t* tests revealed that significant differences were only evident between current and non-self-harmers, and between historic and non-self-harmers, but *not*, as previously mentioned, between current and historic self-harmers. The same was true of anxiety, which was significantly higher in both self-harming groups than in the non-self-harming group. Of course, the implications of these findings are tempered by the fact that depression and anxiety, as measured by the BDI and the BAI, reflect states, not traits: in this case, they reflect emotional state over the last 2 and 4 weeks respectively. State anxiety tends to correlate with trait anxiety [92], but without the inclusion of a measure of trait anxiety, we can only speculate whether trait anxiety is a risk-factor for NSSI. Nevertheless, the data corroborates the association between depression, anxiety and self-injury [73, 93–101], and highlights the risk for self-injury in autistic sufferers of anxiety and depression. The most common functional purpose of self-injury reported by our sample was the regulation of low-energy states (e.g. depression, dissociation), with the second most popular function being the alleviation of high-energy states (which include anxiety). The qualitative data, too, makes mention of depression and anxiety, which are implied by one participant as the cause of self-injury in so far as tackling these issues helps reduce NSSI.

The last variables explored as potential risks for NSSI were autistic traits as a proxy of symptom severity [42], and sensory differences [13, 49]. Interestingly, though both variables were highlighted in the literature on the ‘stereotyped’ form of NSSI often seen in individuals with autism and intellectual disability, sensory sensitivity also appeared in our group as a predictor of group categorisation, and sensory differences were the only variables to predict the range of body areas targeted (sensory avoidance), lifetime incidence of NSSI (sensory avoidance) and the frequency of NSSI in most active phase (sensory low registration). These findings are theoretically consistent with the high level of distress that autistic people report from their sensory disturbances [102–104], and are bolstered by data from our qualitative analysis, where NSSI was linked to being ‘overwhelmed’ by sensory stimulation, and could be helped by learning to identify sensory needs and/or sensory stimulation. That individuals with low registration (that is, under-responsivity to sensory stimulation) might engage in NSSI with higher frequency has alarming implications for injuries more severe than perhaps intended, and highlights self-injury as a potential deleterious association of sensory differences.

### Scientists, loved ones and practitioners: what can we learn from the voices of autistic people?

Improving clinical services and mental health is a highly topical issue and a research priority for the autistic community [105–107], and here special attention should be paid to the voices of autistic people in our qualitative data [105, 108–111]. What implications can be drawn from our qualitative data to inform research and/or clinical practice? As phrased in our question to participants, what should people know if they want to understand and help?

Autistic people in our analysis placed a high importance on understanding the diverse reasons for NSSI, both for the sake of alleviating or controlling their self-injury and so that others could respond to them more appropriately. Furthermore, our analysis demonstrated a need to critically consider the meaning that autistic individuals *themselves* ascribe to NSSI, and we query whether it is the *functions* of NSSI, rather than NSSI in and of itself, which may be predictive of mental illness and suicidality. This relates back to the dichotomy seen between individuals who felt a great deal of distress and helplessness in the face of self-injury, and those who appeared to approach their self-injury quite practically and methodically as a coping mechanism. The framing of self-injury as an ‘addiction’ draws an interesting parallel with research in non-autistic participants and the lay view of self-injury [112]. Some theorists speak of it as a ‘process addiction’ with addictive features including compulsion, loss of control, difficulties stopping and increasing tolerance [113]. Biologically, the pain of injury stimulates the release of endogenous opiates which can produce analgesic and euphoric effects [114]. Others investigating NSSI alongside cravings for and abuse of substances point out that consciously, self-injury is craved for reasons of negative reinforcement (reduction of aversive emotions) rather than by positive reinforcement [115]. This is the kind of negative reinforcement described by one participant who felt ‘calm’, after feeling ‘overwhelmed’, upon seeing blood. Interestingly, non-autistic participants suggest that quite aside from pain, looking at the blood from self-injury seems particularly important for many self-harmers, serving to, likewise, ‘make [s] me feel calm’ [116].

If self-injury is powerfully reinforced by behavioural contingencies, clinicians should be aware that NSSI is not easily changed. However, they must also be aware of the latter group of participants who may see no problem with their self-injury, as a conscious choice that might be preferable to other options and one that might, in fact, cause problems if made unavailable. This view may be somewhat alien to loved ones and clinicians who quite naturally, and rightly considering its links to suicidality, view NSSI with high concern. Of course, where participants appear to express clear distress that they attribute to their self-injury, this clearly necessitates action from the conscientious practitioner. However, we suggest that a measured response, one that acknowledges a potentially functional, ‘rational’ purpose of NSSI, may be beneficial whilst clinicians ascertain the functional role and meaning ascribed to NSSI by that individual.

Both questions highlighted the great importance of how others react to the autistic individual (the need for compassion; empathy; non-judgement; patience; and open-mindedness, avoiding assumptions and emotionality). Participants appeared to place great value on the safety and regularity of the therapist’s office as a place to talk and be heard, as has also been emphasised in the treatment of self-harming non-autistic patients [32, 117]. Several participants highlighted that communication is problematic, that therapists and clinicians may be ‘speaking a different understanding’ (P23) and that misunderstandings can give rise to feelings of despair in individuals who are trying to comply. It is important that clinicians recognise the especial communicative needs of this group, and that likewise, commonly used clinical tools, designed for non-autistic people, may not be entirely fit for purpose [118–121].

Therapeutic goals that autistic adults highlighted as helpful in helping them understand and decrease self-injury included increasing poor self-esteem and low self-confidence, decreasing self-criticism, teaching practical strategies and managing sensory issues. Both low self-esteem [122, 123] and self-criticism [124–127] have been linked to NSSI; poor self-esteem, in particular, appears (along with weak coping strategies) to mediate the relationship between personality pathology and self-injury [128]. Accordingly, therapeutic interventions in non-autistic participants commonly attempt to decrease self-criticism and improve self-worth, alongside teaching more adaptive coping strategies [32, 117, 129, 130]. Teaching more adaptive means of emotional expression and regulation is also an element of these interventions, as is replacing behaviours with alternatives meeting the same functional purposes; this is concordant with our participants highlighting alternative behaviours and damage limitation strategies that are helpful for them in managing self-injury. Our participants also highlighted that identification and management of sensory issues, and understanding their self-injury within the context of their autism, had been helpful. Whether sensory interventions or so-called sensory diets of prescribed activities, the efficacy of which is debated in terms of positive outcomes [131, 132], have any positive effect on autistic NSSI in this population is yet to be determined. As so little research focuses on therapeutic interventions for autistic adults, this is an important avenue for future study.

### Limitations and directions for future study

The present study aimed to advance understanding of NSSI in autistic adults without intellectual disability, an understudied group. Given the relationship of NSSI to mental ill-health and suicidality, an understanding of the particular *risk* factors that might improve identification and treatment of NSSI is a worthwhile goal. Lacking a control group of non-autistic participants, the present study cannot certify whether the variables that predicted classification as current, historic or non-self-harmers—alexithymia, depression, anxiety and sensory sensitivity—are especial risk factors for *autistic* people or would similarly indicate non-autistic individuals at greater risk of self-injury. The small sample of autistic and non-autistic self-harmers compared in previous research [15] imply many similarities in the use of NSSI between groups, but these preliminary findings require validation in a larger group, alongside examination of risk variables for self-injury.

Limitations to the current work include the variables we were unable to consider, notably IQ, which could not be operationalised in this online design. We surmise that our participants would be considered to have an IQ in the average to high range [> 70]); over half were qualified to degree level in each group, and all participants had attended school to GCSE (UK) or equivalent level. As such, our conclusions must be considered with caution as regards their relevance to autistic people *with* intellectual disabilities; although self-injurious behaviour in this group also seems related to sensory differences [13, 49], it remains to be investigated whether alexithymia, for instance, increases the risk of self-injury in autistic individuals with intellectual impairment. Similarly, we were unable to obtain a more thorough operationalization of autistic symptomatology, such as might be provided through use of the Autism Diagnostic Observation Schedule [133], and therefore operationalised this variable only through use of the AQ. Furthermore, given the debate described above regarding the use of RMET to measure theory of mind [91], further investigation of the role of sociocommunication difficulties related to mentalizing in NSSI might be valuable.

Limitations of our statistical approach should also be considered. We adopted an a priori analysis informed by prior literature, and did not correct significance values for multiple statistical tests. The issue of multicollinearity, overlapping variance between measures, is also one to be considered. As can be seen in our analysis and Additional Materials, many of the predictor variables explored in this study tend to correlate: previous literature has demonstrated, for example, that alexithymia is strongly associated with autism and autistic traits [82], that a relationship exists between autistic traits and depressive symptomatology [134] and that depression and anxiety often co-exist in autistic [135, 136] and non-autistic [137] populations. In an analysis of this size, it is hard to identify the unique contribution of any one of these variables, stripped of the overlapping variance with its fellows. The analysis does, however, highlight some screening tests, for example alexithymia, as being more valuable than others if clinicians are concerned about self-injury.

Much remains to be ascertained to understand the incidence of NSSI in autistic adults with *and* without intellectual disability and for identifying those most at risk. Given the divergence in how participants perceive their self-injury, we question the *nature* of the risk, the link between self-injury, suicide ideation and suicidal acts seen in non-autistic people [3–9]. Future research might investigate whether NSSI similarly increases the risk of suicide ideation and behaviours in autistic individuals, or whether this relationship is mediated by another factor/s, such as self-esteem, the function played by NSSI or the perceptions held by participants about it; whether those in whom NSSI might indicate a suicide risk can be identified. Although several variables are highlighted here as potentially important in the aetiology of self-injury, it remains to be ascertained whether, systematically and specifically targeted, alleviation of related symptoms is beneficial to those who suffer from their self-injury.

Another broader query, within this study and pertaining to general clinical use, concerns the use of measurement and assessment tools designed for non-autistic individuals. As previously mentioned, tools specific to autistic individuals are now being developed to accurately assess depression and suicidality [118, 119], but the key measures in this study were designed for use with non-autistic individuals and have never been validated with autistic groups. As one participant phrased so aptly, non-autistic clinicians ‘use their understanding of how they operate to judge an autistic operating system’, and thus are liable to make assumptions about the way psychometric items are perceived or interpreted. A very basic example, from the NSSI-AT, is the continuously used phrase ‘hurt yourself’. The instrument thoroughly assesses methods of self-injury, but at no point queries whether the participant actually experiences *pain* from any of these behaviours. There has been rigorous scientific debate as to whether autistic people have different pain thresholds to non-autistic people, or at least have a qualitatively different subjective experience of it [138–140]. It is plausible that an individual who does not experience pain from NSSI might be perturbed by a literal understanding of the term ‘hurt yourself’. Another instance is the TAS-20, which despite being robustly used in autism research (see, for instance, [80, 82, 141]), has never undergone a thorough examination (e.g. [118, 119]) as to whether autistic individuals understand items in the same way as non-autistic people; the very nature of the alexithymia construct might challenge comprehension of the test. Although our data suggests that the TAS-20 is a reasonable predictor of engagement in NSSI, the fact that this and other measures were not designed with autistic people in mind raises important questions about how the utility of these measures might be maximised for clinical usage.

## Conclusions

The present study attempted to elucidate the features of self-injury in autistic individuals without intellectual disability, and to explore the thoughts of participants regarding their self-injury and helpful interventions. There was great diversity in the quantitative and qualitative data, with several participants signalling the disruptive effects of NSSI, their distress and lack of control over it, whilst others indicated that NSSI appeared to play a functional role in their lives, compartmentalised as a coping technique under their control. Variables that might differentiate self-harming from non-self-harming individuals were also of interest in so far as they might predict the incidence of self-injury and provide vital clues for understanding and treating the phenomenon. Current and historic self-harmers were set apart from non-self-harmers by their scores in measures of depression, anxiety, alexithymia and sensory sensitivity.

## Additional File

Additional file 1: **Table S1.** Pearson correlation coefficients between predictor variables. Table S2 Qualitative analysis: Experiences with therapy. Table S3 Qualitative analysis: what others should know about NSSI. (DOCX 45 kb)

## Abbreviations

ASC: Autism spectrum conditions
NSSI: Non-suicidal self-injury
NSSI-AT: Non-Suicidal Self-Injury Assessment Tool
RMET: Reading the Mind in the Eyes Test

## Footnotes

1 We choose here to use the identity-first language preferred by the majority of autistic individuals [142], but respect the right of individuals to use person-first or identity-first language.

2 We did not ourselves confirm the diagnostic status of participants, but confirmed the precise diagnosis they had received and the date they received it when entering the study.

3 Of note, studies of NSSI in typically-developing (non-autistic) participants suggest that sex differences are especially apparent in the methods that people use to self-injure, with women more likely to engage in cutting specifically [74, 143]. Some studies suggest men more likely to engage in hitting or burning [143].

4 Though, notably, differences could also be partially due to measures used: we employed the Beck Depression Inventory, whilst Maddox and colleagues used the Patient Health Questionnaire-9 [144].
