# Supplementary material for: A ‘choice’, an ‘addiction’, a way ‘out of the lost’: exploring self-injury in autistic people without intellectual disability
Source: Mol Autism. 2019 Apr 11;10:18. doi: 10.1186/s13229-019-0267-3 (PMC6458651; doi:10.1186/s13229-019-0267-3)
Supplement: Supplementary file 1 — Table S1. Pearson correlation coefficients between predictor variables. Table S2 Qualitative analysis: Experiences with therapy. Table S3 Qualitative analysis: what others should know about NSSI. (DOCX 45 kb) [file 13229_2019_267_MOESM1_ESM.docx]

Supplementary Materials

1. Supplementary Table 1: Correlations between predictor variables
2. Supplementary Table 2: Qualitative analysis: experiences with therapy
3. Supplementary Table 3: Qualitative analysis: what other people should know about NSSI
4. Supplementary Table 1: Pearson correlation coefficients between predictor variables

|  | AQ | BDI | BAI | Alexithymia | RMET | Sensory low registration | Sensory seeking | Sensory sensitivity | Sensory avoidance |
| --- | --- | --- | --- | --- | --- | --- | --- | --- | --- |
| AQ |  | r =.245,  p = .019 | r =.178,  p = .09 | r =.238,  p = .02 | r = -.05,  p = .65 | r =.380,  p < .001 | r = -.048,  p = .65 | r =.521,  p < .001 | r =.529,  p < .001 |
| BDI | r = 245,  p = .019 |  | r = .629,  p < .001 | r = .414,  p < .001 | r = .024,  p = .82 | r = .249,  p = .01 | r = -.143,  p = .15 | r = .276,  p = .005 | r = .149,  p = .13 |
| BAI | r =.178,  p = .09 | r = .629,  p < .001 |  | r = .370,  p < .001 | r = .04,  p = .70 | r = .328,  p = .001 | r = -.158,  p = .11 | r = .501,  p < .001 | r = .304,  p = .002 |
| Alexithymia | r =.238,  p = .02 | r = .414,  p < .001 | r = .370,  p < .001 |  | r = .282,  p = .006 | r = .424,  p < .001 | r = .284,  p = .004 | r = .365,  p < .001 | r = .267,  p = .006 |
| RMET | r = -.05,  p = .65 | r = .024,  p = .82 | r = .04,  p = .70 | r = .282,  p = .006 |  | r = -.061,  p = .56 | r = .140,  p = .18 | r = .026,  p = .8 | r = -.05,  p = .63 |
| Sensory low registration | r = .380,  p < .001 | r = .249,  p = .01 | r = .328,  p = .001 | r = .424,  p < .001 | r = -.061,  p = .56 |  | r = .278,  p = .004 | r = .667,  p < .001 | r = .494,  p <.001 |
| Sensory seeking | r = -.048,  p = .65 | r = -.143,  p = .15 | r = -.158,  p = .11 | r = .284,  p = .004 | r = .140,  p = .18 | r = .278,  p = .004 |  | r = -.012,  p =.9 | r = -.009,  p = .93 |
| Sensory sensitivity | r =.521,  p < .001 | r = .276,  p = .005 | r = .501,  p < .001 | r = .365,  p < .001 | r = .026,  p = .8 | r = .667,  p < .001 | r = -.012,  p =.9 |  | r = .796,  p < .001 |
| Sensory avoidance | r =.529,  p < .001 | r = .149,  p = .13 | r = .304,  p = .002 | r = .267,  p = .006 | r = -.05,  p = .63 | r = .494,  p <.001 | r = -.009,  p = .93 | r = .796,  p < .001 |  |

Supplementary Table 1: Pearson correlation coefficients (r) depicting correlations between each of the predictor variables included in the primary analysis. Significant correlations (uncorrected for multiple comparisons) are shaded grey.

1. Supplementary Table 2: Qualitative analysis: Experiences with therapy

“**What** **in your experience with therapy** (even if your intentionally hurting yourself was not the focus of your therapy) **has been most helpful** **in helping you to understand or control intentionally hurting yourself?”**

Participant numbers are in brackets, with ‘c’ denoting a current and ‘h’ denoting a historic self-harmer.

| Themes | Subthemes | Quotations |
| --- | --- | --- |
| Understanding myself |  | “Why I would do it.” (P24,c)  “The therapies that have been most effective have focused of dealing with the causes of this problem rather than the problem itself.” (P33,c) |
|  | Emotional awareness:   - *Understanding the cause of emotions* - *Identifying emotions* - *Expressing emotions* - *Emotions in control* - *Management strategies* | “Understanding what makes me stressed out and what my sensory issues are, and checking in with myself every few hours to know if I am hungry/too hot/too cold/thirsty/tired is helpful because my problems often happen when I have very high stress and I'm in some immediate situation. If I keep my stress levels low then I won't be as frustrated, so I won't have those kind of meltdowns.” (P39,c)  “better understanding my anger and the causes of it (not necessarily any relevant, suggested coping mechanisms/strategies) was the most constructive in regards to self-injury.” (P12,c)  “…starting to understand my emotions and what is 'upsetting’.” (P72,h)  “Understanding and tackling the causes of depression and anxiety” (P71,h)  “Verbalising some feelings so that I can understand them better.” (P17,c)  “learning and understanding emotions” (P48,c)  “I haven't attended therapy for over a decade, but better understanding my anger and the causes of it (not necessarily any relevant, suggested coping mechanisms/strategies) was the most constructive in regards to self-injury” - P12  “Learning to name my thoughts and feelings and not letting them take control over me.” (P11,c)  “Verbalising some feelings so that I can understand them better.” (P17,c)  “That I hurt myself out of …fear of showing confusing emotions or being aggressive towards the others …” (P74,h)  “Reading and speaking to other people openly” (P72,h)  “[Understanding] that I hurt myself out of [...] fear of showing confusing emotions or being aggressive towards the others” (P74,h)  “Verbalising some feelings so that I can understand them better.” (P17,c)  “Learning to control stress.” (P15,c)  “Anger control” (P69,h)  “not letting them [thoughts and feelings] take control over me.” (P11,c)  “I exercised more control with myself in terms of exercise or hobbies whenever I felt out of control.” (P64,h)  “…how I change my feelings in a positive way, i.e. go for a walk or paint something.” (P72,h)  “Learning that I have other ways to change how I feel - or don't feel - and learning to do those things.” (P22,c)  “I found ways to stop letting my emotional pain build up to such a level that I couldn't cope with it without a release… These were all repair strategies I was given to stop my cyclic thought behaviour.” (P64,h)  “Having regular time where I was expected to check in with my emotions. If I don't have that time, things creep up on me without me realizing.” (P27,c)  “Learning that I have other ways to change how I feel - or don't feel - and learning to do those things.” (P22,c)  “Use other techniques to deal with frustration.” (P42,c)  “Learning strategies to deal with stressed thoughts.” (P2,c)  “Learning to control stress.” (P15,c)  “Learning other ways to calm myself through MBSR training.” (P76,h) |
|  | Sensory issues | “Understanding what makes me stressed out and what my sensory issues are, and checking in with myself every few hours to know if I am hungry/too hot/too cold/thirsty/tired is helpful because my problems often happen when I have very high stress and I'm in some immediate situation. If I keep my stress levels low then I won't be as frustrated, so I won't have those kind of meltdowns.” (P39,c)  “My therapist did not attempt to stop me from self-harm but almost encouraged it, so it's not a fair assessment. We did however explore options of generating strong sensory input without causing injury (similar to 'skills lists' for BPD).” (P5,c) |
|  | Self-esteem | “Understanding self-worth in responsibility.” (P3,c)  “To try to build my self-esteem.” (P51,h)  “Anger control and building self-confidence.” (P69,h)  “Acknowledging the past actions and not being judged.” (P75,h)  “That I hurt myself out of low self-love and low self-esteem (which did improve in time, and so did self-injuries become less possible), and also out of fear of showing confusing emotions or being aggressive towards the others (which improved by my decision to stop 'limiting myself' and partly by learning about my ASC”. (P74,h) |
|  | Co-morbidities | “Understanding and tackling the causes of depression and anxiety” (P71,h)  “The OCD and anxiety meds and using artificial nails so I cannot scratch hard or tear at myself. Like glueing canoe paddles to my fingers but helps a lot.” (P13,c)  “Controlling my eating disorder.” (P58,h) |
|  | ASC diagnosis | “My diagnosis of Asperger's Syndrome.” (P61,h)  “Being told it was just part of my frustrations of being autistic.” (P73,h)  “Actually having my ASD diagnosis has been the most helpful thing, CAT therapy was good, CBT made me much much worse.” (P10,c)  “Discussing the then current situation - this was with a psychologist during the ASC assessment.” (P37,c)  “That I hurt myself out of low self-love and low self-esteem (which did improve in time, and so did self-injuries become less possible), and also out of fear of showing confusing emotions or being aggressive towards the others (which improved by my decision to stop 'limiting myself' and partly by learning about my ASC)”. (P74,h) |
| Practical strategies |  | “I found ways to stop letting my emotional pain build up to such a level that I couldn't cope with it without a release. I exercised more control with myself in terms of exercise or hobbies whenever I felt out of control. These were all repair strategies I was given to stop my cyclic thought behaviour.” (P64,h)  “Learning that I have other ways to change how I feel - or don't feel - and learning to do those things.” (P22,c)  “Use other techniques to deal with frustration.” (P42,c)  “Learning other ways to calm myself through MBSR training” (P76,h)  “Practical ways to stop, such as using a rubber band, drawing in red etc.” (P53,h)  “My therapist did not attempt to stop me from self-harm but almost encouraged it, so it's not a fair assessment. We did however explore options of generating strong sensory input without causing injury (similar to 'skills lists' for BPD).” (P5,c)  “Reading and speaking to other people openly and starting to understand my emotions and what is 'upsetting', how I change my feelings in a positive way, i.e. go for a walk or paint something.” (P72,h)  “Limiting alcohol if planning to self-harm.” (P44,c)  “The OCD and anxiety meds and using artificial nails so I cannot scratch hard or tear at myself. Like glueing canoe paddles to my fingers but helps a lot.” (P13,c) |
| Specific forms of therapy |  | “Actually having my ASD diagnosis has been the most helpful thing, CAT therapy was good, CBT made me much much worse.” (P10,c)  “CBT.” (P48,h)  “Psychotherapy.” (P68,h)  “Talking with my consultant occupational therapist.” (P32,c)  “Learning other ways to calm myself through MBSR training.” (P76,h) |
| Relationships |  | “Having someone who understood and could help with what was distressing me.” (P62,h)  “Having regular time where I was expected to check in with my emotions. If I don't have that time, things creep up on me without me realizing.” (P27,c)  “Acknowledging the past actions and not being judged.” (P75,h)  “Nothing's really helped with it, it's my way to cope. That being said, talking to a therapist may occasionally remove the need to hurt myself.” (P41,c)  “Discussing the then current situation - this was with a psychologist during the ASC assessment.” (P37,c)  “I see this (NSSI) as a smaller part of the bigger issue and so talking to the therapist about the bigger issue helps, and so reduces the frequency. (P16,c)  “Relationship with the therapist, being heard, learning and understanding emotions” (P48,c)  “Understanding how much I mean to other people (family) and that they want to support me even if I am deeply distressed.” (P45,c)  “Therapy was helpful but when alone for periods in my life, I will regress to self-harming” (P8,c)  “Reading and speaking to other people openly and starting to understand my emotions and what is 'upsetting', how I change my feelings in a positive way, i.e. go for a walk or paint something.” (P72,h) |
| Psychotherapy is not beneficial for everyone |  | “Actually having my ASD diagnosis has been the most helpful thing, CAT therapy was good, CBT made me much much worse.” (P10,c)  “Therapy not understand autistic person they use their understanding of how they operate to judge an autistic operating system so all it does is give confusion they get cross and I feel sad and lost because I am not being good and compliant.” *(*P23,c)  “Nothing it was totally inappropriate and traumatised me sitting in a group and hearing terrible stories.” (P46) |

1. Supplementary Table 3: Qualitative analysis: what others should know about NSSI

**“Finally, what do you think is important to know if people want to understand and help those who intentionally hurt themselves?”**

Participant numbers are in brackets, with ‘c’ denoting a current and ‘h’ denoting a historic self-harmer.

| Themes | Subthemes | Quotations |
| --- | --- | --- |
| Understand the reason |  | We have a reason to do this. Don't judge.” (P15,c)  “To understand the root cause.” (P20,c)  “It comes in part from a reason, that has to be solved and is different for everyone, but in time becomes a routine, how to dis-manage problems in life, a bit like addiction.” (P72,h)  Why they're doing it. You can't hope to tackle the problem without dealing with the root cause.” (P33,c)  “That it's just the symptom of a significant problem.” (P41,c)  “What the root cause of it is.” (P54,h)  “The outer wound only hints at a much more painful inner (hidden) wound.” (P59,h)  “Why they thinking the way they are.” (P48,h)  “So instead of focussing on the symptom (harming myself) rather look at the cause (stress, high pressure) and work with mindfulness and relaxation strategies.” (P21,c)  “Ask the setting; provocations; feelings induced by self-harm.” (P73,h) |
|  | Emotional pain and stress | “Oftentimes it is because of frustration, or feeling like you are stuck in a situation, or simply being too overwhelmed by sensory input, or people not listening to you when you asked for an environmental change.” (P39,c)  “Self-harming helps me to function. Some days things happen and I feel so overwhelmed that I feel like I will break completely and I have no idea how I can get through my day (I work). Seeing the blood is like flipping a switch and I am suddenly completely calm and quite dissociated, numb, everything goes away for a bit whilst I just watch my wounds.” (P3,c)  “… usually when I have to do something stressful that I don't want to do, like a job interview or long boring meetings with people. I can either not do the act or cause some pain to achieve homeostatic balance. Self injury help(s) reduce stress so I could engage with others in appropriate manner.” (P7,c)  “When distressed it has helped me be able to face my loved ones again. Often prior to self-harming I feel so distressed I cannot bear to be seen even by them or to face their smiles.” (P1,c)  “That we are not crazy, it is a coping mechanism to convert emotional pain into physical pain.” (P9)  “That sometimes, if controlled appropriately, it can be a helpful way to control overwhelming feelings.” (P16,c)  “… When I do it there is something in my head I really want to get rid of, usually a horrible feeling of shame, and the self-harming gets rid of it and I feel relieved.” (P18,c)  “Unresolved emotions especially anger.” (P67)  “For me, biting myself and harming myself is a strategy that works for minimizing pressure inside. In periods without stress I don't hurt myself.” (P21,c)  “It is not attention seeking behaviour it's usually because they are unable to cope with their own emotions or feelings, or lack outlets to funnel their periods of stress or anxiety/inability to cope.” (P62,h)  “That it's not to seek attention, it's an attempt to self-regulate or cope with overwhelming emotions or find brief relief from suffering.” (P28,c)  “Mine started very young, slapping biting and hitting. It was never a thing I did consciously, I would be 'upset' or confused and knee jerk reaction was to do that.” (P70,h)  “Hurting yourself does not need to have a reason, frustration is enough.” (P36,c)  On top of that having verbalizing difficulties about my feelings makes it much worse because I know what I want to say but I cannot communicate that to other people in stressful situations, so it's like a positive feedback loop. The more frustrated I am, the less I can talk, and the less I can talk, the more frustrated I am. And eventually that builds up and it feels like I need to do something to get rid of it. And that's where the self-harming comes in for me.” (P39,c)  “That for some of us, it's better than most alternatives and it shouldn't be considered altogether negative. There might be better ways to deal with emotions, but understanding that it can be a positive thing could prove helpful in teaching people those better ways.” (P41,c)  “Empathy, understand how traumatic it can be if you cannot communicate normally, cannot regulate your emotions and feel isolated or stigmatised etc. when you know that inside you are a rational and sentient being.” (P45,c) |
|  | Low self-esteem | “That it is about self-punishment in some cases and that it is a compulsion, NOT a choice!” (P26,c)  “I hurt myself because of my self-hatred and desire to punish myself for the problems I have with everything due to my Aspergers. I would like people to understand that Aspergers people aren’t the way they are intentionally, and they don’t like being the way they are, but aren’t in control of it. Please be nice!!!” (P47,c) |
|  | The question of choice   - *A lack of choice and control* - *A conscious choice…*   *… and should not always be seen as a negative thing* | “That it happened just like that; I had no control over hurting myself.” (P6,c)  “It is difficult for me to say because I really don't know why I did it. I would say that it was definitely not a conscious decision for me, it is a sign of how unwell I was.” (P17,c)  “That it is about self-punishment in some cases and that it is a compulsion, NOT a choice!” (P26,c)  “In time becomes a routine, how to dis-manage problems in life, a bit like addiction.” (P72,h)  Relief and compulsion and I have no other sincere words to describe my irrational actions.” (P65,h)  “That it's a choice, the scars stay with you for life and its an addiction like anything helps - but it's a better option than suicide.” (P46,c)  “It makes me worry about one of my children who has ASC, and how can I help her if I cannot fully control myself?” (P45,c)  “Not everyone who self harms has an impulse control problem. I am not impulsive, my self harm is compulsive. I can resist almost overwhelming urges for months, and only give in when I am very worn down by it...” (P27,c)  “That sometimes there is no choice, they are doing it to get through life and because no one has given them a suitable alternative” (P10,c)  “They don’t want to do it.” (P44,c)  “Mine started very young, slapping biting and hitting. It was never a thing I did consciously, I would be 'upset' or confused and knee jerk reaction was to do that. I didn't always know what I was doing until someone had held my hands down or stopped me in some way and calmed the situation.” (P70,h)  “I have no problem with intentional hurting. I know when and why. Usually when I have to do something stressful that I don't want to do, like a job interview or long boring meetings with people. I can either not do the act or cause some pain to achieve homeostatic balance. Self injury help(s) reduce stress so I could engage with others in appropriate manner. People need to know that it is often the external world that is causing stress to them, not themselves. They need to know that they may choose to hurt themselves because they cannot escape or walk way from stressful situations.” (P7,c)  “I think it is important to know that self-harm, much like any creative or artistic outlet, is a form of expression that some people turn to when words or other communicative methods do not fully convey how they feel...” (P12,c)  “That it's a choice, the scars stay with you for life and its an addiction like anything helps - but it's a better option than suicide.” (P46,c)  “That sometimes, if controlled appropriately, it can be a helpful way to control overwhelming feelings…” (P16,c)  “People often assume that those who self harm are drama queens or irresponsible and overly emotional. Actually when I self-harm it is very private, and in 'normal' life I'm very quiet and calm and responsible…” (P18,c)  “For me, biting myself and harming myself is **a strategy that works** for minimizing pressure inside. In periods without stress I don't hurt myself.” (P21,c)  “Not everyone who self harms has an impulse control problem. I am not impulsive, my self harm is compulsive. **I can resist** almost overwhelming urges for months, and only give in when I am very worn down by it...” (P27,c)  “Oftentimes it is because of frustration, or feeling like you are stuck in a situation, or simply being too overwhelmed by sensory input, or people not listening to you when you asked for an environmental change. For me it's often due to frustration or lack of control on my life, plus extra everyday stress on top of it, or just feeling defeated and like there's no way I can change a situation. On top of that having verbalizing difficulties about my feelings makes it much worse because I know what I want to say but I cannot communicate that to other people in stressful situations, so it's like a positive feedback loop. The more frustrated I am, the less I can talk, and the less I can talk, the more frustrated I am. And eventually that builds up and **it feels like I need to do something to get rid of it. And that's where the self-harming comes in for me.”** (P39,c)  “Reducing the stigma/mindset that it's a terrible. Of course there are different degrees of self-harm, but mine is fairly mild and has **simply helped me to cope with certain situations**. To me it's almost a positive thing, but I am concerned that people in the public will be shocked if they see someone biting their arm or banging their head.” (P5,c)  “That for some of us, it's better than most alternatives and it shouldn't be considered altogether negative. There might be better ways to deal with emotions, but understanding that it can be a positive thing could prove helpful in teaching people those better ways.” (P41,c)  “That sometimes, if controlled appropriately, it can be a helpful way to control overwhelming feelings. As long as it is controlled and isn't causing huge degrees of harm, then there could be many worse things the person could be doing.” (P16,c) |
|  | Individuality | “It's really important to find out how to address each individual, there is a common misconception that we all fit in the same box.. we really don't.” (P70,h)  “I think every person is different, so not to assume anything.” (P58,h)  “That there are many reasons, not everyone wants to talk about it but try reaching out and being non-judgemental.” (P60,h) |
|  | Unknown causes | “The person doing the harm will not know why they are doing it. There's no simple answer.” (P11,c)  “It is difficult for me to say because I really don't know why I did it. I would say that it was definitely not a conscious decision for me, it is a sign of how unwell I was.” (P17,c)  “That they might not realise they are doing it.” (P25,c) |
| Challenge your assumptions |  | “That we are not crazy, it is a coping mechanism to convert emotional pain into physical pain.” (P9)  “People often assume that those who self harm are drama queens or irresponsible and overly emotional. Actually when I self-harm it is very private, and in 'normal' life I'm very quiet and calm and responsible…” (P18,c)  “It is not attention seeking behaviour it's usually because they are unable to cope with their own emotions or feelings, or lack outlets to funnel their periods of stress or anxiety/inability to cope.” (P62,h)  “That it's not to seek attention, it's an attempt to self-regulate or cope with overwhelming emotions or find brief relief from suffering.” (P28,c)  “People often assume that those who self harm are drama queens or irresponsible and overly emotional. Actually when I self-harm it is very private, and in 'normal' life I'm very quiet and calm and responsible…” (P18,c)  “Not everyone who self-injures does it for attention. When medical personnel are treating these people, they deserve the same respect and treatment that anyone else would get. Don't assume they 'like pain' and refuse them anaesthetic.” (P22,c)  “Not everyone who self harms has an impulse control problem. I am not impulsive, my self harm is compulsive. I can resist almost overwhelming urges for months, and only give in when I am very worn down by it. It's quite offensive when people then assume it's impulsive or attention-seeking. It's a part of my autism - a repetitive, ritualistic, stereotyped behaviour that has developed with me for the past 20 years. Not everyone who self harms has a personality disorder!” (P27,c)  “We are hurting inside - not seeking attention.” (P69,h)  “Not everyone just does it for attention.” (P71,h)  “That it's not attention-seeking and does not automatically mean that the person has borderline personality disorder.” (P50,h) |
| NSSI serves a function | A coping mechanism | “Self-harming helps me to function. Some days things happen and I feel so overwhelmed that I feel like I will break completely and I have no idea how I can get through my day (I work). Seeing the blood is like flipping a switch and I am suddenly completely calm and quite dissociated, numb, everything goes away for a bit whilst I just watch my wounds. Then I can go back and get on with meetings and talking to people. I bandage the wounds up so that the blood won’t get on my clothes but the pain of it (a secret pain that I can press on) also keeps me grounded.” (P3,c)  “When distressed it has helped me be able to face my loved ones again. Often prior to self-harming I feel so distressed I cannot bear to be seen even by them or to face their smiles.” (P1,c)  “I have no problem with intentional hurting. I know when and why. Usually when I have to do something stressful that I don't want to do, like a job interview or long boring meetings with people. I can either not do the act or cause some pain to achieve homeostatic balance. Self injury help(s) reduce stress so I could engage with others in appropriate manner.” (P7,c)  “That we are not crazy, it is a coping mechanism to convert emotional pain into physical pain.” (P9)  “That sometimes, if controlled appropriately, it can be a helpful way to control overwhelming feelings.” (P16,c)  “… When I do it there is something in my head I really want to get rid of, usually a horrible feeling of shame, and the self-harming gets rid of it and I feel relieved.” (P18,c)  “For me, biting myself and harming myself is a strategy that works for minimizing pressure inside. In periods without stress I don't hurt myself.” (P21,c)  “It is not attention seeking behaviour it's usually because they are unable to cope with their own emotions or feelings, or lack outlets to funnel their periods of stress or anxiety/inability to cope.” (P62,h)  “That it's not to seek attention, it's an attempt to self-regulate or cope with overwhelming emotions or find brief relief from suffering.” (P28,c)  “What I did was very mild, just taking off the skin which caused my skin in that area to harden. If anyone had tried to get me to stop, I would have been much, much worse. I am told I cannot talk about my feelings, although I believe I do. I was not aware of the behaviour being related to any feeling and any treatment may have caused more harm than good.” (P63,h)  “Reducing the stigma/mindset that it's a terrible. Of course there are different degrees of self-harm, but mine is fairly mild and has simply helped me to cope with certain situations.” (P5,c)  “Hurting yourself does not need to have a reason, frustration is enough. In addition, it could be classified as a self-management technique, such as alcohol or drugs.” (P36,c) |
|  | Self-expression | “I think it is important to know that self-harm, much like any creative or artistic outlet, is a form of expression that some people turn to when words or other communicative methods do not fully convey how they feel.” (P12,c) |
| Respond appropriately | Calm, non-judgment and compassion | “We have a reason to do this. Don't judge.” (P15,c)  “Never get emotional about it with someone, it doesn't help and will likely make them retreat further so as to not cause worry.” (P10,c)  “That they cannot stop it happening, it's not their fault and they can only support the person doing it.” (P19,c)  “Not to get angry or condescending with them for self-harming. This is counterproductive.” (P31,c)  “I think it is important to know that self-harm, much like any creative or artistic outlet, is a form of expression that some people turn to when words or other communicative methods do not fully convey how they feel. Giving people the tools to understand the why and the how of their emotions and understanding them rather than judging them is a very powerful gift.” (P12,c)  “Empathy.” (P68,h)  “Empathy, understand how traumatic it can be if you cannot communicate normally, cannot regulate your emotions and feel isolated or stigmatised etc. when you know that inside you are a rational and sentient being. I think that ASC is the loneliest way of being unless you are fortunate enough to have empathy and understanding from the people around you.” (P45,c)  “Please be nice!!!” (P47,c)  “To be patient and understanding. Non-judgemental and considerate.” (P49,h)  “Important to know that I am not alone in thinking about and actually harming myself. Many do it.” (P14,c)  “It is very important so that they don't feel alone. I live on my own and no-one notices my injuries unless I point it out.” (P40,c)  “People need relationships, love and appreciation.” (P56,h)  “Be there for them. Make sure they know they're loved. DON'T leave them alone. My problems worseneed because I was alone and lonely. I'm surprised I'm still alive. Absolutely make sure the person self-harming knows they can get through it. Only mention professional help once, but bring it up gently.” (P53,c)  “That they can get help.” (P64,h)  “That there are many reasons, not everyone wants to talk about it but try reaching out and being non-judgemental.” (P60,h)  “Ask the setting; provocations; feelings induced by self-harm.” (P73,h) |
|  | Acknowledge the role of autism | “That if you are not autistic your rules and expectations are not the same. You are speaking a different understanding and it is so hard to find a moment where understanding touches.” (P23,c)  “To not beat around the bush. Just try and speak openly about it.” (P24,c)  “Empathy, understand how traumatic it can be if you cannot communicate normally, cannot regulate your emotions and feel isolated or stigmatised etc. when you know that inside you are a rational and sentient being. I think that ASC is the loneliest way of being unless you are fortunate enough to have empathy and understanding from the people around you.” (P45,c)  “… having verbalizing difficulties about my feelings makes it much worse because I know what I want to say but I cannot communicate that to other people in stressful situations, so it's like a positive feedback loop. The more frustrated I am, the less I can talk, and the less I can talk, the more frustrated I am. And eventually that builds up and it feels like I need to do something to get rid of it. And that's where the self-harming comes in for me.” (P39,c)  “I hurt myself because of my self-hatred and desire to punish myself for the problems I have with everything due to my Aspergers. I would like people to understand that Aspergers people aren’t the way they are intentionally, and they don’t like being the way they are, but aren’t in control of it. Please be nice!!!” (P47,c) |
